# A structural approach for finding functional modules from large biological networks

**DOI:** 10.1186/1471-2105-9-S9-S19

**Published:** 2008-08-12

**Authors:** Mutlu Mete, Fusheng Tang, Xiaowei Xu, Nurcan Yuruk

**Affiliations:** 1 Department of Applied Science, University of Arkansas at Little Rock, Little Rock, Arkansas, USA; 2 Department of Biology, University of Arkansas at Little Rock, Little Rock, Arkansas, USA; 3 Department of Information Science, University of Arkansas at Little Rock, Little Rock, Arkansas, USA

## Abstract

**Background:**

Biological systems can be modeled as complex network systems with many interactions between the components. These interactions give rise to the function and behavior of that system. For example, the protein-protein interaction network is the physical basis of multiple cellular functions. One goal of emerging systems biology is to analyze very large complex biological networks such as protein-protein interaction networks, metabolic networks, and regulatory networks to identify functional modules and assign functions to certain components of the system. Network modules do not occur by chance, so identification of modules is likely to capture the biologically meaningful interactions in large-scale PPI data. Unfortunately, existing computer-based clustering methods developed to find those modules are either not so accurate or too slow.

**Results:**

We devised a new methodology called SCAN (Structural Clustering Algorithm for Networks) that can efficiently find clusters or functional modules in complex biological networks as well as hubs and outliers. More specifically, we demonstrated that we can find functional modules in complex networks and classify nodes into various roles based on their structures. In this study, we showed the effectiveness of our methodology using the budding yeast (Saccharomyces cerevisiae) protein-protein interaction network. To validate our clustering results, we compared our clusters with the known functions of each protein. Our predicted functional modules achieved very high purity comparing with state-of-the-art approaches. Additionally the theoretical and empirical analysis demonstrated a linear running-time of the algorithm, which is the fastest approach for networks.

**Conclusion:**

We compare our algorithm with well-known modularity based clustering algorithm CNM. We successfully detect functional groups that are annotated with putative GO terms. Top-10 clusters with minimum p-value theoretically prove that newly proposed algorithm partitions network more accurately then CNM. Furthermore, manual interpretations of functional groups found by SCAN show superior performance over CNM.

## Background

Biological systems can be modeled as complex network systems with many interactions between the components. These interactions give essential information about the function and behavior of the analyzed system. For example, the protein-protein interaction network is the physical basis of multiple cellular functions. The first large-scale protein interaction studies were conducted on yeast [1,2], followed by more recent studies on the fly [3] and the worm [4]. The main goal of current network research in many diverse areas such as biology, social sciences and statistical physics is to understand and characterize underlying network structures. Such an understanding is vital for many systems, simply because *structure always affects function *[5].

As noted by Barabasi [6], the most important discovery of network research in recent years is that many different complex network systems including biological networks demonstrate some significant common principles that govern their architecture, topology and behavior such as small-world property [7,8], power-law degree distribution [7] and highly modular structures [9].

Among other network analysis tasks network clustering plays a particular role as it helps us to detect modules or communities which are usually good indicators of structural or functional units of the underlying network.

Various methods have been developed to find partitions in networks. These methods tend to partition networks such that there are a dense set of edges within every partition and few edges between partitions. Modularity-based algorithms [10-12] and normalized cut [13,14] are widely used examples. However, most of the state-of-the-art algorithms have quadratic running time thus limited usage for large real networks.

Recently we proposed a new network clustering algorithm, SCAN (Structural Clustering Algorithm for Networks) which runs linearly with the size of given network [15]. Despite the common methodology of current methods where maximization or minimization of edges within/across clusters is essential, it defines clusters based on structural similarity of vertices. Number of shared neighbors for two vertices basically defines their similarity, and vertices with similarity values above certain threshold are assigned to the same partition. Similarity definition that takes into account the neighborhood of a vertex becomes more reasonable when social networks are considered: people who share more friends are more likely to be friends each other and more likely to be members of same community.

Additionally its capability to detect hubs and isolated nodes in the given network makes it a unique approach as no other available methods offer such a function.

In this study, we show the effectiveness of our methodology using the budding yeast (Saccharomyces cerevisiae) protein-protein interaction network. To validate our clustering results, we compare our clusters with the known functions of each protein. Our predicted functional modules achieve very high clustering scores as compared to other state-of-the-art approaches.

## Results and discussion

### Protein-Protein Interaction Network

Hand-curated databases of PPI in *Saccharomyces cerevisiae *have been studied earlier in the literature [16-18] and are proven to be invaluable resources for bioinformatics research. For this study, PPI network is downloaded from the *Saccharomyces *Genome Database (SGD) [19] on January 21, 2008. After cleaning unrelated interaction, we chose only Affinity Capture-MS and Affinity Capture-Western proteins, which account for 26751 interactions between 4030 proteins.

### Validation metric based on Gene Ontology

The Gene Ontology (GO) database provides controlled vocabularies for the description of the 1) molecular function, 2) biological process, and 3) cellular component of gene products. The ontologies are continuously updated by GO Consortium, and new versions are made available on monthly basis. Of three ontologies, molecular function describes the tasks performed by individual gene products, such as enzyme activator activity and RNA binding; biological process refers broad biological goals, such as chromatin remodeling or mRNA capping; and cellular component covers subcellular structures, locations, and macromolecular complexes, such as intracellular or cytoplasm.

The ontologies of GO database are manually created by many scientists. GO database is accepted as ground-truth and used for comparison and validation purposes. Thus, in our analysis we used GO ontologies to test if the resulting clusters are related to any known functional modules. Simply relying on number of proteins that have same annotation will be misleading since distributions of genes among various GO categories are not uniform.

P-value is the probability that a given set of proteins is enriched by a given functional group by random chance. It is usually used as a criteria to assign each cluster to a known function [20,21]. The smaller the p-value, the more evidence the clustering is not random. In terms of GO annotations, a group of genes with smaller p-value is more significant than the one with a higher p-value.

Consider a cluster with size n, m proteins sharing a particular annotation *A*. Also assume that there are *N *proteins in the PPI database, and *M *of them are known to have annotation *A*. Given that, the probability of observing m or more proteins that are annotated with *A *out of *n *protein is:

$$p-value=\frac{\left(\begin{array}{c} M\\ i \end{array}\right)\left(\begin{array}{c} N-M\\ n-i \end{array}\right)}{\left(\begin{array}{c} N\\ n \end{array}\right)}$$

Based on above formulation, p-value is calculated for each of three ontologies. However we cannot find always three p-values for a cluster since it is not guaranteed that each cluster has at least one member associated with each of ontology. For instance, protein trm2 does not have any association for cellular component, whereas protein mms1 has two entries which are both from biological process ontology. Assuming a cluster has only two members, trm2 and mms1, we cannot calculate a p-value of the cluster for cellular component ontology. Therefore, it would be correct to claim that we calculate at least one p-value for each cluster. In the case of multiple annotations from same ontology, the one with the smaller p-value is assigned to the cluster as functional annotation. That being said, the p-value without any restriction is not enough to label clusters as significant. Hence we use the recommended cutoff value of *0.05 *in order to select significant clusters within each ontology.

Let *C *be a cluster including k annotations, and *A*^*C *^denote proteins having annotation *A *in cluster *C*. The cluster $C=\{A_{1}^{C}\cap A_{2}^{C}\cap ...\cap A_{k}^{C}\}$ is labeled with a functional annotation $A_{t}^{C}$, 1 ≤ *t *≤ *k*, iff p-value of $A_{t}^{C}$ is the smallest one among others in cluster *C *and less than cutoff value. After all, we call a cluster insignificant if it has no functional annotation.

**Figure 1:**
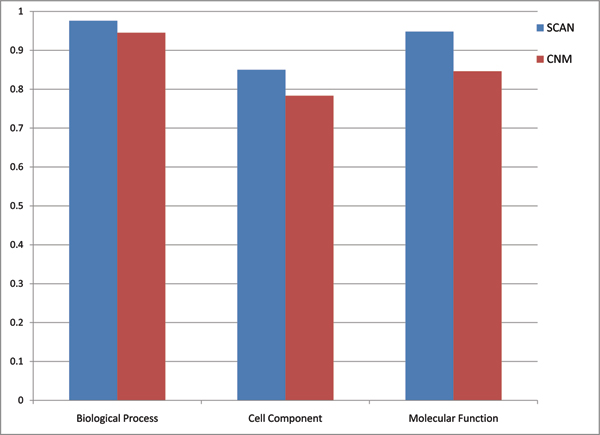
Comparison of clustering scores for three GO categories.

While functional annotations, backed by the statistical evidence, are good interpretation for a single cluster, they do not have much impact to quantify the overall clustering accuracy. Therefore we employ a measure called clustering score [22] to compare two clustering layouts.

$$Clustering\;Score=1-\frac{\sum_{i=1}^{n_{s}}\min(p_{i})+(n_{i}*cutoff)}{(n_{i}+n_{s})*cutoff},$$

where *n*_*s *_and *n*_*i *_denotes the number of significant and insignificant clusters, respectively and min(p_*i*_) represents the smallest p-value of a significant cluster. Note that *min(p*_*i*_*) *equals to the p-value of functional annotation at the same time. Clustering score is calculated for three different categories of the GO Ontology, molecular function, biological process, and cell component. In Figure 1 clustering scores are shown for SCAN and CNM. Please refer Additional file 1 for annotation of each cluster.

Furthermore, to show how SCAN clearly outperforms CNM, we listed top-10 clusters having the smallest p-values with corresponding GO categories in Table 1 and Table 2. For the category of biological process, SCAN finds clusters with smaller p-values. Also note that p-values of clusters in SCAN are increasing gradually from first to tenth cluster (4.45E-98 to 9.29E-28). In contrast, CNM results start with greater p-value (2.10E-61) and spot clusters with larger size. Recall that the smaller p-value is the better to annotate a cluster with certain function. However, some clusters of CNM with smaller p-values are still hard-to-interpret because of their enormous size, such as cluster 14 having 220 proteins, cluster 12 with 919 proteins, and cluster 48 with 549 proteins.

**Table 1:** Top-10 SCAN clusters with highest p-values

	**Cluster ID**	**P -value**	**GO Term**	**Term Freq. in Network**	**Term Freq. in Cluster**	**Cluster Size**
**Biological Process**	1	4.45E-98	nuclear mrna splicing, via spliceosome	66	58	88
	89	1.01E-65	translation	252	58	64
	5	1.16E-52	ubiquitin-dependent protein catabolic process	60	34	56
	2	9.04E-40	transcription from rna polymerase ii promoter	50	41	288
	15	8.58E-38	anaphase-promoting complex-dependent proteasomal ubiquitin-dependent protein catabolic process	13	13	13
	22	1.36E-30	chromatin remodeling	46	20	40
	192	5.46E-29	vacuolar acidification	23	13	16
	13	6.36E-29	chromosome segregation	36	16	25
	24	2.14E-28	regulation of microtubule polymerization or depolymerization	10	10	12
	30	9.29E-28	regulation of cell growth	10	10	13

**Cellular Components**	7	6.81E-53	cytosolic large ribosomal subunit	80	55	222
	89	1.50E-51	mitochondrial small ribosomal subunit	33	29	64
	1	2.53E-41	u4/u6 × u5 tri-snrnp complex	27	25	88
	15	9.01E-36	anaphase-promoting complex	15	13	13
	22	7.15E-31	rsc complex	16	15	40
	24	2.14E-28	dash complex	10	10	12
	38	2.14E-28	trapp complex	10	10	12
	185	7.18E-26	ribonuclease mrp complex	9	9	11
	155	5.84E-25	smc5-smc6 complex	8	8	8
	53	4.93E-24	dna replication preinitiation complex	21	12	22

**Molecular Functions**	89	5.64E-71	structural constituent of ribosome	210	58	64
	5	2.75E-45	endopeptidase activity	26	24	56
	1	7.12E-45	contributes_to rna splicing factor activity, transesterification mechanism	29	27	88
	37	6.44E-41	snap receptor activity	24	23	74
	192	4.65E-28	hydrogen ion transporting atpase activity, rotational mechanism	12	11	16
	185	7.18E-26	contributes_to ribonuclease mrp activity	9	9	11
	22	6.60E-23	contributes_to dna-dependent atpase activity	15	12	40
	34	1.30E-22	protein transporter activity	24	14	46
	15	2.25E-18	ubiquitin-protein ligase activity	44	10	13
	8	1.37E-17	dolichyl-diphosphooligosaccharide-protein glycotransferase activity	8	8	35

**Table 2:** Top-10 CNM clusters with highest p-values

	**Cluster ID**	**P -value**	**GO Term**	**Term Freq. in Network**	**Term Freq. in Cluster**	**Cluster Size**
**Biological Process**	15	2.10E-61	nuclear mrna splicing, via spliceosome	66	55	220
	17	5.00E-40	transposition, rna-mediated	33	19	22
	13	5.11E-31	rna elongation from rna polymerase ii promoter	53	51	919
	49	4.30E-25	ribosomal large subunit assembly and maintenance	39	34	549
	16	1.09E-23	anaphase-promoting complex-dependent proteasomal ubiquitin-dependent protein catabolic process	13	13	75
	58	2.78E-22	microtubule nucleation	22	16	98
	53	7.86E-20	trna processing	14	8	10
	45	8.35E-18	negative regulation of gluconeogenesis	9	7	12
	56	3.15E-17	ubiquitin-dependent protein catabolic process via the multivesicular body pathway	13	11	91
	63	1.73E-13	mrna polyadenylation	17	13	241

**Cellular Components**	11	3.67E-66	mitochondrial large ribosomal subunit	43	41	107
	49	4.72E-42	cytosolic large ribosomal subunit	80	64	549
	17	5.00E-40	retrotransposon nucleocapsid	33	19	22
	58	1.28E-30	condensed nuclear chromosome kinetochore	30	22	98
	16	2.48E-24	anaphase-promoting complex	15	14	75
	20	1.58E-23	peroxisomal membrane	12	9	11
	37	9.63E-23	smc5-smc6 complex	8	8	11
	53	2.36E-22	ribonuclease mrp complex	9	8	10
	63	5.21E-18	mrna cleavage and polyadenylation specificity factor complex	14	14	241
	65	3.13E-15	alpha-1,6-mannosyltransferase complex	6	6	18

**Molecular Functions**	17	1.71E-26	rna binding	130	19	22
	58	2.34E-24	structural constituent of cytoskeleton	47	22	98
	53	2.36E-22	contributes_to ribonuclease mrp activity	9	8	10
	13	9.16E-19	dna-directed rna polymerase activity	31	30	919
	49	6.52E-17	snorna binding	21	20	549
	48	5.97E-13	contributes_to protein transporter activity	7	5	10
	30	1.24E-10	nad-independent histone deacetylase activity	4	4	15
	65	2.79E-10	contributes_to alpha-1,6-mannosyltransferase activity	4	4	18
	33	1.34E-09	endopeptidase activity	26	24	1408
	63	2.52E-09	contributes_to histone lysine n-methyltransferase activity (h3-k4 specific)	7	7	241

For molecular function, similar to biological process, there is a significant difference between two algorithms in terms of both p-values and size of clusters. While SCAN clusters have p-values between 5.64E-71 and 1.37E-17 and average cluster-size of 44, CNM yields clusters with p-values ranging between 1.71E-26 and 2.52E-09 and average size of 329 (10 to 1408). Size problem for molecular function seems even worse than biological process.

In the category of cellular component, group of top-10 clusters starts with cluster 10, p-value 3.67E-66, size of 107. It is good start against SCAN, however, p-values of CNM do not show regular increase as seen in SCAN clusters. Additionally, regarding CNM results, fluctuation in clustering size arises once again and makes the evaluation intricate. Thus, we randomly picked a few clusters and analyzed the accuracy manually.

### Validation based on manual comparisons

To judge the significance of a cluster, we manually analyzed whether the function of each member corresponds to cluster's assigned function from three different GO Ontologies, biological processes, cellular components, and molecular functions. We chose cluster sizes ranging from 10 to 30 members since most functional complexes contain the comparable numbers of protein components.

**Figure 2:**
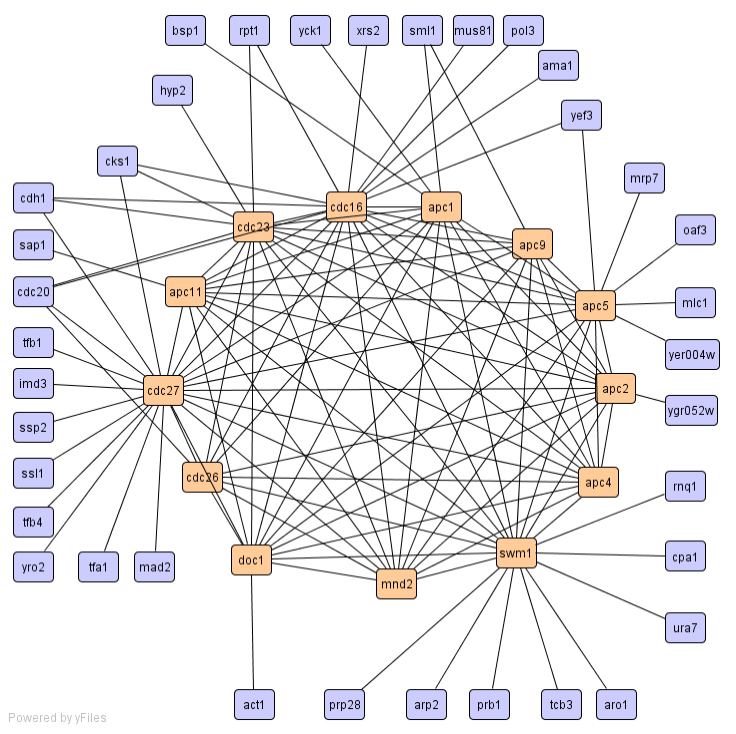
Cluster of anaphase promoting complex proteins.

For biological process, SCAN assigned all the anaphase promoting complex proteins (apc1; apc11; apc2; apc4; apc5; apc9; cdc16; cdc23; cdc26; cdc27; doc1; mnd2; swm1) into one cluster. The clustering result is shown in Figure 2. In this graph, interactions only for cluster members are shown to keep the network visually readable. Proteins that are members of found cluster are denoted in different color. Although SCAN identified most subunits of the APC/C complex, it missed a few key components such as cdc20 and cdh1. This is due to the multi-valent interactions of these proteins. SCAN assigned these two proteins in the hub category. Unlike SCAN, CNM merged these proteins into a big cluster with 75 members, which also includes proteins involved in translation initiation, ergosterol synthesis, and other cellular processes. SCAN also identified the vacuolar H+- ATPase complex (rav1, rav2, vma10, vph1, etc) and grouped them into a cluster with 16 members. In this cluster, there are three proteins that have not been reported to directly function in the assembly of the vacuolar H+-ATPase. Xdj1p is a chaperone-like protein. Yig1p is involved in anaerobic glycerol production, which may indirectly affect the cytosolic proton homeostasis. Ymr027wp is a protein with unknown function so far. From our clustering results, we predict that xdj1p and ymr027wp are also involved in the assembly of the vacuolar H+-ATPase. Contrary to the SCAN results, CNM grouped these vacuolar H+-ATPase proteins into a huge cluster with 1416 members, which is not insightful for any predictions.

**Figure 3:**
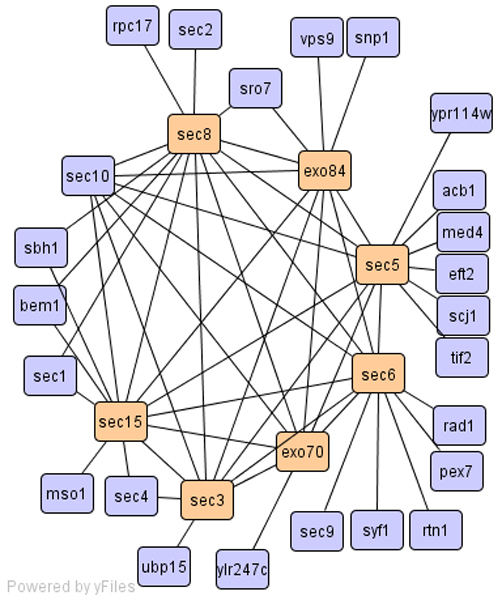
Cluster of exocyst complex.

For cellular components, SCAN accurately identified the exocyst complex (exo70, exo84, sec3, sec15, etc, please refer Figure 3), and the DNA replication preinitiation complex (cdc45, dpb11, mcm10 etc). On the contrary, CNM put the exocyst complex into a group with 551 members and the DNA replication preinitiation complex into a group with 919 members. Although SCAN missed protein Sld2p in the DNA replication preinitiation complex and included two extra proteins (srp101p, srp102p), SCAN predicted a new function for erv2p. Erv2p is an ER lumen protein required for the formation of disulfide bonds. Our clustering result suggests that erv2p helps the proper folding of the proteins required for DNA replication. Both SCAN and CNM detected the peroxisome membrane protein complex (SCAN cluster-176 with 9 members; CNM cluster-19 with 11 members). Please refer Additional file 2 and 3 for detailed clustering results. The two extra proteins (pex3, pex19) in CNM cluster 19 are also members of the peroxisomal membrane complex.

**Figure 4:**
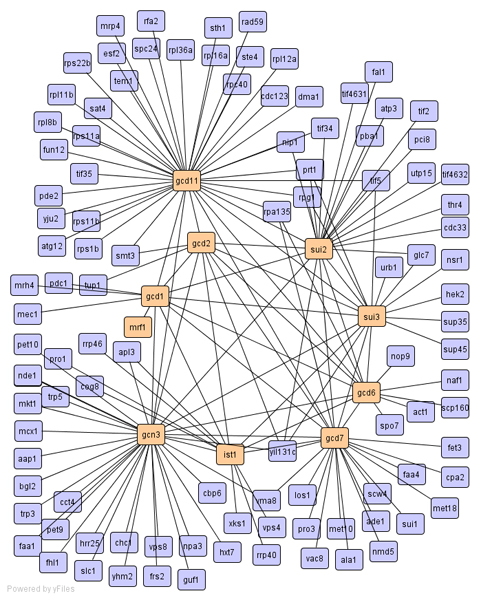
Cluster of translation initiation complex.

For molecular functions, SCAN found translation initiation complex (cluster-105 with 10 members; gcd1, gcd11, ist1, mrf1, etc). The found clustering result is depicted in Figure 4. CNM again assigned these proteins into a huge cluster with 1416 members. The function of protein glycosylation is achieved by many proteins in multiple subcellular locations including the lumen of ER and golgi. After glycosylation, the proteins will be transported to their right locations by secretion or other sub-cellular transport systems. SCAN assigned 35 proteins to one cluster. This cluster contains the oligosaccharyltransferase complex proteins (ost1, ost2, ost3, etc) of the ER lumen, the golgi mannosyltransferase complex proteins (mnn9, mnn10, mnn11), and secretion proteins (sec61, sec62, sec63, etc). Grouping these proteins into one cluster emphasizes the potential of applying SCAN in analysis of dynamic biological networks.

With regard to assigning proteins with similar functions to a cluster, SCAN out-weighted CNM in 6 out of 7 clusters we analyzed manually. Moreover, smaller size clusters found by SCAN enable us to predict the function of each cluster more accurately.

## Methods

### The notion of structure-connected clusters

Our goal is to achieve an optimal clustering of the PPI network, as well as to identify hubs and outliers. Therefore, both connectivity and local structure are used in our definition of optimal clustering. In this section, we formulize the notion of a structure-connected cluster, which extends that of a density-based cluster [23] and can distinguish good clusters, hubs, and outliers in networks. In the next section, we present, SCAN, an efficient algorithm to find the optimal clustering of networks [15].

### Structure-connected clusters

The existing network clustering methods are designed to find optimal clustering of networks based on the number of edges that run within or across clusters. Direct connections are important, but they represent only one aspect of the network structure. We believe that the neighborhood around two connected vertices is also important. The neighborhood of a protein includes all the vertices connected to it by an edge. When you consider a pair of connected vertices, their combined neighborhood reveals neighbors common to both vertices.

**Figure 5:**
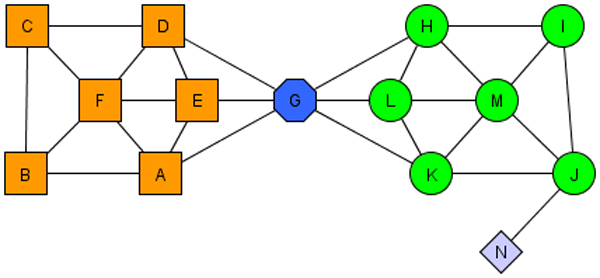
A small network demonstrating two clusters, a hub (vertex 6), and an outlier (vertex 13).

Our method is based on common neighbors. Two vertices are assigned to a cluster according to how they share neighbors. This makes sense when you consider social communities. People who share many friends create a community, and the more friends they have in common, the more intimate the community. But in social networks there are different kinds of actors. There are also people who are outsiders (like hermits), and there are people who are friendly with many communities but belong to none (like politicians). The latter plays a special role in small-world networks as *hubs *[24]. Such a hub is illustrated by protein *G *in Figure 5.

More formally, we focus on simple, undirected and unweighted graphs. Let *G *= {*V*, *E*} be a graph, where *V *is a set of vertices -proteins in PPI; and *E *is set of unordered pairs of distinct vertices, called edges. Before formal presentation of SCAN, it is worth to give fundamental definitions exploited by the algorithm. We refer the reader to our previous work [15] for extensive formal discussions.

The structure of a vertex is described by its neighborhood. A formal definition of vertex structure is given as follows.

#### Definition 1 (Vertex Structure)

Let *v ∈ V*, the structure of v is defined by its neighborhood, denoted by

*Γ (v) = {w ∈ V | (v, w) ∈ E} ∪ {v}*

Please note that neighborhood of protein *v, Γ (v)*, also includes v in addition to all neighbors of v. For instance, considering Figure 5, *Γ (A) *would be *{A, B, E, F, G}*. Having Definition 1, now we can formulize similarity function, which is run for every edge, *{v, w} ∈ E*, in the network. We call the similarity function structural similarity because it is solely derived from vertex structure *Γ (v)*. The structural similarity between two vertices is measured by normalized common neighbors, which is also called cosine similarity measure commonly used in information retrieval. If we only use the number of shared neighbors, hub vertices, such as *G *in Figure 5, will be clustered into either of the clusters or two clusters will be mistakenly merged. Therefore, we normalize number of common neighbors by the geometric mean of the two neighborhoods' size. Note that In Figure 5, protein *G *should be identified as a hub, shared in neighborhood of both clusters.

#### Definition 2 (Structural Similarity)

$$\sigma (v,w)=\frac{\left|\Gamma (v)\cap \Gamma (w)\right|}{\sqrt{\left|\Gamma (v)||\Gamma (w)\right|}}$$

When a member of cluster shares a similar structure with one of its neighbors, their computed structural similarity will be large. Obviously structural similarity is symmetric, *σ (v, w) = σ (w, v)*. Structural similarity between *v *and *w, σ (v, w)*, would be greater than zero if and only if v and w are vertices of an edge *e ∈ E*. Under this circumstance, structural similarity attains values between (0, 1]. However, structural similarity should be restricted to control expansion of the cluster. Therefore, we apply a threshold *ε *to the computed structural similarity when assigning cluster membership, formulized in the following *ε*-neighborhood definition.

#### Definition 3 (*ε*-Neighborhood)

*N*_*ε *_(*v*) = {*w *∈ *Γ *(*v*)|*σ *(*v*, *w*) ≥ *ε*}

When a vertex accumulates enough neighbors in its *ε*-neighborhood, it becomes a nucleus or *seed *for a cluster. Such a vertex is called a core vertex. Core vertices are a special class of vertices that have a minimum of *μ *neighbors with a structural similarity that greater than or equals to the threshold *ε*. From core vertices we grow the clusters. In this way only the parameters *μ *and *ε *determine the clustering of networks. For a given *ε*, the minimal size of a cluster is determined by *μ*. If a vertex w is in *ε*-neighborhood of a core vertex *v*, vertex *w *should be included into the same cluster with vertex *v*. Because, they are connected and share a similar structure. This concept is known as *direct structural reachability*.

Direct structural reachability is symmetric for any pair of cores. However, it is asymmetric if one of the vertices is not core. Also the property of direct structural reachability is basis for the cluster expansion. A newly formed cluster C consists of a core vertex v and v's *ε*-neighborhood. Then we try to expand cluster C through any vertex w in v's *ε*-neighborhood. This approach guarantees that vertex w is directly structure-reachable from vertex v. Iterative queries for direct structural reachability usually add more and more vertices into the current cluster. This procedure mimics a chain effect for core vertices.

**Figure 6:**
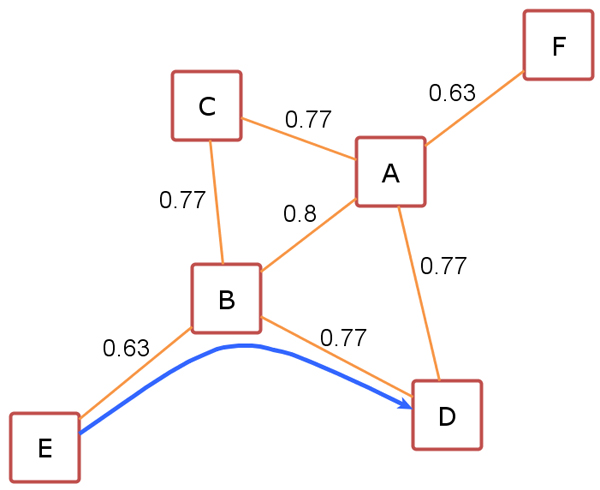
A toy network demonstrating structural reachability. Similarities between vertices are given.

A simple setting is shown in Figure 6. Under the conditions of *μ *= *2 *and *ε *= *0.6*, the possible scenario is as follows: We find *E *as the first core vertex since structural similarity between *E *and *B *is greater than *ε, 0.6*. Now a cluster of *{E, B} *is formed, and it should be expanded if possible. At the second step we look for any vertex that is similar to *B*. Among neighbors of *B*, vertex *D *is selected and inserted into the current cluster *{E, B} *due to similarity value of *0.77 *between *B *and *D*. After the insertion, the cluster has now three vertices *{E, B, D}*. At this stage of algorithm, it is noticeable that vertex *E *and *B *are core vertices; *B *is directly structure-reachable from *E*; and *D *is directly structure-reachable from *B*.

After given example, we introduce another property of SCAN algorithm: structural reachability, which can be considered as chained form of direct structural reachability. The structural reachability is transitive, but it is asymmetric. It is only symmetric for a pair of cores, as appears in previous example. More specifically, the structural reachability is a transitive closure of direct structural reachability.

Two non-core vertices in the same cluster may not be structure-reachable because the core condition may not hold for them. But they still belong to the same cluster because they both are structure- reachable from the same core. This idea is known as structural connectivity, and explained more formally as follows. A vertex *v ∈ V *is structure-connected to a vertex *w ∈ V *w.r.t *ε *and *μ*, if there is a vertex *u ∈ V *such that both v and w are structure-reachable from *u*. The structural connectivity is a symmetric relation. For the structure-reachable vertices, it is also reflective. Now we are ready to define a cluster as structure-connected vertices, which is maximal w.r.t. structural reachability.

A non-empty subset *C *⊆ *V *is called a structure-connected cluster w.r.t *ε *and *μ*, if all vertices in *C *are structure-connected and *C *is maximal w.r.t structure reachability. The SCAN algorithm finds all clusters w.r.t *ε *and *μ*, however, there might be some isolated vertices that are not assigned to clusters. If this is the case, we categorize each of those vertices either as hub or outlier. When an isolated vertex *v ∈ V *has neighbors belonging to two or more different clusters, it is labeled as hub vertex. Otherwise, isolated vertex would be an outlier.

In practice, the definitions of a hub and an outlier are flexible. It may be more useful to regard hubs as a special kind of outlier, since both are isolated vertices. The more clusters in which an outlier has neighbors, the more strongly that vertex acts as a hub between those clusters. This point will be discussed further when we consider actual networks.

### Algorithm scan

In this section, we describe the algorithm SCAN which implements the search for clusters, hubs and outliers in PPI network. The search begins by first visiting each vertex once to find structure-connected clusters, and then visiting the isolated vertices to identify them as either a hub or an outlier.

**Figure 7:**
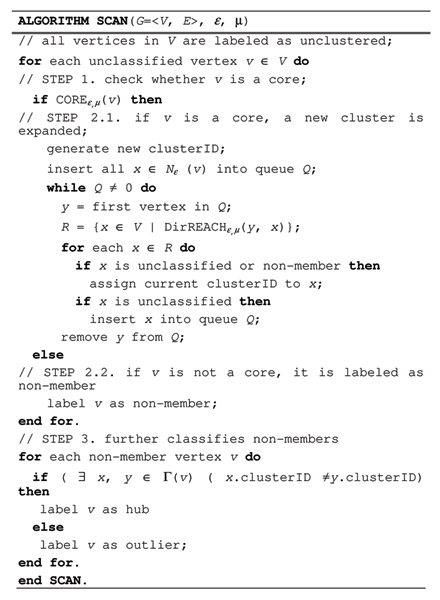
Pseudocode of SCAN Algorithm.

**Figure 8:**
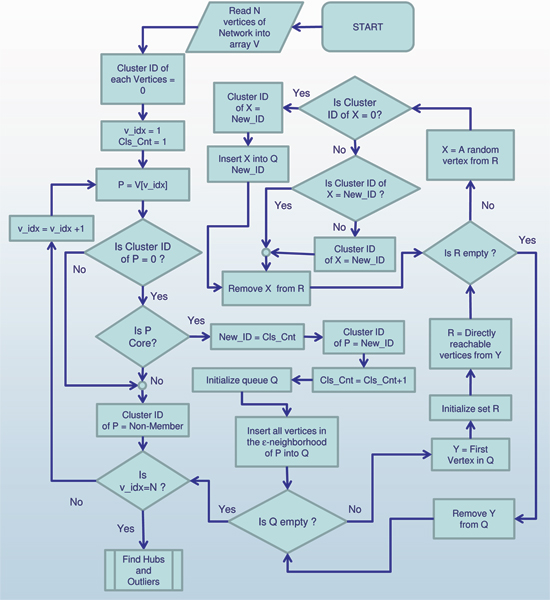
Graphic diagram of the main body of algorithm SCAN.

**Figure 9:**
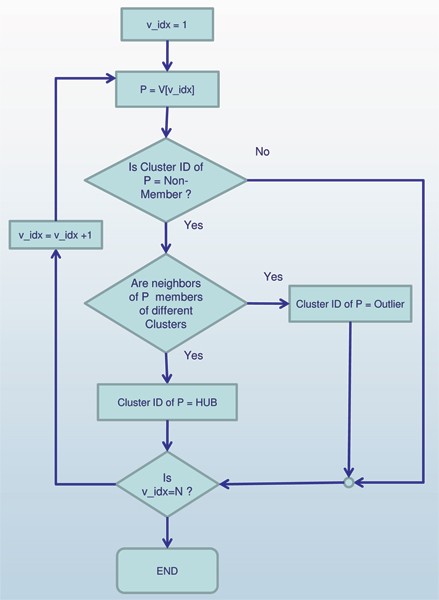
Graphic diagram of Find Hubs and Outliers, a subprocedure of algorithm SCAN.

The pseudo code of the algorithm SCAN is presented in Figure 7 with graphical representation in Figure 8 and Figure 9. SCAN performs one pass of a network and finds all structure-connected clusters for a given parameter setting. At the beginning all vertices are labeled as unclassified. The SCAN algorithm classifies each vertex either a member of a cluster or a non-member. For each vertex that is not yet classified, SCAN checks whether this vertex is a core (STEP 1). If the vertex is a core, a new cluster is expanded from this vertex (STEP 2.1). Otherwise, the vertex is labeled as a non-member (STEP 2.2). To find a new cluster, SCAN starts with an arbitrary core *v *and search for all vertices that are structure-reachable from *v *in STEP 2.1. This is sufficient to find the complete cluster containing vertex *v*, due to given definitions. In STEP 2.1, a new cluster ID is generated which will be assigned to all vertices found in STEP 2.1. SCAN begins by inserting all vertices in *ε*-neighborhood of vertex *v *into a queue. For each vertex in the queue, it computes all directly structure-reachable vertices and inserts those vertices into the queue which are not yet classified. This is repeated until the queue is empty.

The non-member vertices can be further classified as hubs or outliers in STEP 3. If an isolated vertex has edges to two or more clusters, it is classified as a hub. Otherwise, it is an outlier. This final classification is done according to what is appropriate for the network. As mentioned earlier, the more clusters in which an outlier has neighbors, the more strongly that vertex acts as a hub between those clusters. Likewise, a vertex might bridge only two clusters, but how strongly it is viewed as a hub may depend on how aggressively it bridges them.

As discussed before, the results of SCAN do not depend on the order of processed vertices, i.e. the obtained clustering of network (number of clusters and association of cores to clusters) is determinate.

### Complexity analysis

In this section, we present an analysis of the computation complexity of the algorithm SCAN. Given a graph with *m *edges and *n *vertices, SCAN first finds all structure-connected clusters w.r.t. a given parameter setting by checking each vertex of the graph (STEP 1). This entails retrieval of all the vertex's neighbors. Using an adjacency list, a data structure where each vertex has a list of which vertices it is adjacent to, the cost of a neighborhood query is proportional to the number of neighbors, that is, the degree of the query vertex. Therefore, the total cost is *O(deg(v*_1_*)+deg(v*_2_*)+...deg(v*_*n*_*))*, where *deg(v*_*i*_*), i = 1,2,..., n *is the degree of vertex *v*_*i*_. If we sum all the vertex degrees in *G*, we count each edge exactly twice: once from each end. Thus the running time is *O(m)*.

We also derive the running time in terms of the number of vertices, should the number of edges be unknown. In the worst case, each vertex connects to all the other vertices for a complete graph. The worst case total cost, in terms of the number of vertices, is *O(n(n-1))*, or *O(n2)*. However, real networks generally have sparser degree distributions. In the following we derive the complexity for an average case, for which we know the probability distribution of the degrees. One type of network is the random graph, studied by Erdös and Rényi [25]. Random graphs are generated by placing edges randomly between vertices. Random graphs have been employed extensively as models of real world networks of various types, particularly in epidemiology. The degree of a random graph has a poisson distribution:

$$p(k)=\left(\begin{array}{c} n\\ k \end{array}\right)p^{k}{\left(1-p\right)}^{n-k}\approx \frac{z^{k}e^{z}}{k!}$$

which indicates that most nodes have approximately the same number of links (close to the average degree *E(k) = z*). In the case of random graphs the complexity of SCAN is *O(n)*.

Many real networks, such as social networks, biological networks and the WWW follow a power-law degree distribution. The probability that a node has *k *edges, *P(k)*, is on the order k-*α*, where *α *is the degree exponent. A value between 2 and 3 was observed for the degree exponent for most biological and non-biological networks studied by the Faloutsos brothers [26] and Barabási and Oltvai [5]. The expected value of degree is *E(k) = α/(α-1)*. In this case the average cost of SCAN is again *O(n)*.

Therefore, the complexity in terms of the number of edges in the graph for SCAN algorithm is in general linear. The complexity in terms of the number of vertices is quadratic in the worst case of a complete graph. For real networks like biological networks, social networks, and computer networks, SCAN expects linear complexity with respect to the number of vertices as well.

## Conclusion and research directions

We devised a new methodology called SCAN (Structural Clustering Algorithm for Networks) that can efficiently find clusters or functional modules in complex biological networks as well as hubs and outliers [15]. We showed the effectiveness of our methodology using the budding yeast (Saccharomyces cerevisiae) protein-protein interaction network.

To validate our clustering results, we compared our clusters with the known functions of each protein. Additionally, we compared our algorithm with well-known modularity based clustering algorithm, CNM [12]. We successfully showed that SCAN can detect functional groups that are annotated with GO terms. Top-10 clusters with minimum p-values demonstrated that clusters of the newly proposed algorithm are more accurate than those of CNM. Manual interpretations of functional groups found by the new algorithm also showed superior performance over CNM.

Furthermore, a computational complexity analysis demonstrated a linear running-time of the algorithm, which makes it, to our knowledge, the fastest approach for finding clusters in networks.

## Competing interests

The authors declare that they have no competing interests.

## Authors' contributions

XX and FT have conceived the study. XX is the inventor of the algorithm SCAN used for this study. NY and MM developed the software and performed data analysis, algorithm testing, and benchmarking. FT interpreted clustering results found by the above mentioned algorithms. MM, FT, XX and NY wrote the manuscript.

## Supplementary Material

Additional File 1 Cluster annotations for SCAN and CNM. Click here for file

Additional File 2 Clustering result of CNM for PPI network. Click here for file

Additional File 3 Clustering result of SCAN for PPI network. Click here for file
