# Supplementary material for: A structural approach for finding functional modules from large biological networks
Source: BMC Bioinformatics. 2008 Aug 12;9(Suppl 9):S19. doi: 10.1186/1471-2105-9-S9-S19 (PMC2537570; doi:10.1186/1471-2105-9-S9-S19)
Supplement: Additional File 1 — Cluster annotations for SCAN and CNM. [file 1471-2105-9-S9-S19-S1.pdf]

HUBS, 392 Proteins

aac3 abp1 acc1 aco1 acs2 ade3 adh2 adh3 adr1 aha1 ahp1 ald6 apm1 arb1 arc1 aro1 asc1 asf1 asr1 atg11 atg12 atp2 atp3 bbc1 bem2 bmh1 bmh2 bre5 bsp1 bud14 bud3 bud32 caf4 car2 cbk1 cbr1 cct3 cct5 cct8 cdc13 cdc14 cdc19 cdc20 cdc28 cdc37 cdc55 cdc6 cdc60 cdc7 cdh1 chk1 cks1 cla4 clb2 clb5 cln2 clu1 cmd1 cmp2 cns1 cof1 cpa2 cpr6 cpr7 crn1 csm3 cyc8 cyr1 cys4 dbf2 dbp2 dbp8 ded1 ded81 def1 dia2 dma1 dnl4 dpb2 dun1 ecm10 ede1 eft1 eft2 egd2 ela1 erg13 erp2 ess1 est1 faa4 far1 fas1 fet4 fks1 fpr1 fpr3 gcn1 gcn20 gdh2 gdi1 gfa1 glc7 glt1 gnd1 gpa2 gph1 gre2 grr1 gus1 hap2 hef3 hek2 hem13 hmo1 hog1 hor2 hrp1 hrr25 hrt1 hsc82 hsl1 hsp104 hsp26 hsp42 hsp60 hsp82 hxt7 hyp2 idh1 idh2 ils1 ilv1 ilv2 imd2 imd3 imd4 ino4 inp52 ipp1 kap104 kap122 kap123 kap95 kar2 kel1 kin2 ksp1 kss1 las17 lcd1 lsg1 lsm12 lsp1 lte1 lys12 lys20 mag1 mag2 mcd1 mdh1 mec1 met10 met18 mgm101 mgt1 mis1 mkk2 mkt1 mlc1 mms22 mrd1 mre11 mrpl22 msn5 mus81 myo1 myo2 nam7 nap1 nat1 net1 new1 nhp6a nmd3 nmd5 nop53 nop6 npa3 npl3 nsr1 ntg1 nup42 oaf1 ola1 oye2 pab1 pbp4 pbs2 pds5 pfk1 pfk2 pgi1 pgm2 pho2 pho4 pil1 pma1 pmt1 pol2 pol30 por1 prb1 prp43 prs3 pse1 psh1 psk1 ptc3 ptc6 pub1 pwp1 rad1 rad10 rad16 rad26 rad3 rad4 rad50 rad51 rad53 rad59 rad6 rfa1 rho1 rim1 rim11 rnq1 rnr2 rnr3 rnt1 rom2 rpl40a rpp2a rps0a rps0b rps17a rps25a rps26b rrb1 rsp5 rtg2 rtt101 rtt107 rvs161 rvs167 sac6 sah1 sap155 sap185 sar1 sat4 sba1 scc4 scp160 scs2 sdo1 sds22 sec10 sec2 sec27 sec4 sec53 sec7 sen15 ses1 sgn1 sgv1 shm2 sir2 sir3 sir4 sit4 skt5 slc1 sld2 slt2 slx4 slx5 slx9 smi1 smk1 sml1 smt3 snu13 sod1 spa2 spc1 spo12 sps1 spt2 srp1 srs2 srv2 ssa3 ssa4 ssb2 ssc1 ssd1 sse1 ssz1 ste20 sti1 stm1 sua7 sui1 sup35 sup45 swh1 swi5 sxm1 tat1 tcb3 tcp1 tdh1 tdh2 tdh3 tef1 tef4 tem1 tfa1 tgl5 tif1 tif2 tma19 tom1 top1 top2 tps1 trp3 trx1 trx2 tsc13 tsr2 tub1 tub2 tup1 uba1 ubi4 ubp14 ubp15 ufd4 ura2 ura7 vac8 vip1 vps13 vps4 yak1 yal027w yar010c ybl071w-a ybr159w yck1 ycr043c ydj1 ydr128w yef3 yel023c ygr054w ygr250c yhb1 yhr033w yil131c yil161w yjr138w yku70 yku80 ylr455w yml131w ymr031c ynk1 ynl313c yol087c yol098c yor1 ypl216w ypr102c ypt6 yra1 yrb2 zuo1

OUTLIERS, 1511 Proteins

aac1 aah1 aap1 aat2 abz2 ace2 acf2 acf4 ach1 acm1 aco2 ade1 ade13 ade2 ade4 ade5,7 ade6 ade8 adh4 adh5 adh6 adk1 ado1 ady3 aep1 aep2 aep3 afr1 agx1 ahc1 ahc2 ai1 aip1 akl1 akr2 ala1 ald4 alf1 alg13 alg14 alg3 alg5 alt2 aly1 ama1 amd1 amn1 anb1 aos1 apa1 ape2 ape3 apn2 app1 apt1 ara1 ara2 are1 arf3 arg1 arg3 arg4 arg5,6 arg82 ark1 arl1 arl3 aro2 aro3 aro4 aro9 ase1 asf2 ask10 asn1 asn2 ate1 atf2 atg10 atg20 atg26 atg4 atp16 atp22 atp5 atp7 ats1 aus1 avt2 avt4 ayr1 ayt1 azr1 bag7 bar1 bas1 bat1 bat2 bck1 bck2 bdf2 bdh1 bdh2 bdp1 bem3 bem4 bet2 bet4 bfr1 bgl2 bio2 bio3 bna1 bna3 bna5 bni1 bni4 bni5 bnr1 boi1 boi2 bop3 bph1 bpl1 bpt1 brf1 brl1 bro1 bsc2 bsd2 bst1 bud2 bud23 bud6 bul1 bul2 bur2 bur6 bzz1 caf120 caf16 caf20 caj1 cak1 car1 cat8 cbf1 cbf2 cbp1 cbp2 cbp3 cbp4 cbp6 cca1 ccc2 cce1 cch1 ccs1 cdc123 cdc15 cdc21 cex1 cha1 chs1 chz1 cin1 cit2 cki1 clg1 cln1 cln3 cmk2 coq10 cos111 cox11 cox20 coy1 cpa1 cpr5 crp1 crt10 crz1 cse1 csf1 csn12 csr1 cst26 cst6 ctf4 ctp1 ctr1 ctr3 cue3 cue4 cup2 cus2 cwc24 cwc27 cwh41 cwp2 cyb5 cyc1 cys3 dak2 dal3 dal7 dal81 dbf20 dbp1 dbp5 dcd1 dcg1 dcr2 dcw1 dfm1 dfr1 dia1 dia4 dip5 djp1 dld2 dld3 dma2 dmc1 dnf1 dog1 dog2 dom34 dos2 dot1 dpb3 dpm1 dps1 dre2 dse1 dse4 dsf2 dss1 dtd1 dtr1 dur1,2 dut1 dys1 eap1 ebs1 ecm1 ecm21 ecm22 ecm25 ecm3 ecm30 ecm31 ecm33 ecm37 ecm38 ecm5 edc2 egt2 eht1 elf1 elg1 elm1 emi2 emp46 emp47 emp70 eno1 ent1 ent2 eps1 erf2 erg1 erg20 erg26 erg6 erg9 eri1 ero1 erp4 erv29 esc1 esp1 etr1 exg1 exo1 faa2 fab1 fal1 fap1 fap7 far10 far3 far7 far8 fas2 fbp1 fcf2 fcy1 fcy21 fes1 fig2 fig4 fin1 fir1 fkh2 flc2 fmp46 fol2 fol3 fps1 fre3 frq1 frs1 frs2 frt2 fun14 fun19 fun30 fur1 fus2 fyv4 fyv6 fyv8 gaa1 gac1 gad1 gal10 gal2 gal3 gal7 gal80 gas1 gas5 gat1 gcd10 gcd14 gcr1 gcr2 gcs1 gcv3 gcy1 gda1 gde1 gdh3 gds1 gdt1 gef1 gem1 gfd1 gfd2 gic1 gic2 gip1 gip2 gip3 gip4 gis4 glc8 gle1 glk1 gln3 glo2 glo4 gly1 gna1 gnd2 gpd1 gpd2 gpg1 gpi10 gpi15 gpr1 grc3 grs1 grx1 grx5 gsc2 gsh1 gto1 gto3 gtt2 gtt3 gua1 gud1 guk1 gut1 gvp36 gwt1 gyl1 gyp5 gyp7 gzf3 haa1 hac1 hal5 ham1 hap1 hbs1 hch1 hem1 hem12 hem15 hfa1 hfd1 hfm1 hgh1 hho1 hip1 his3 his4 hlr1 hmf1 hmg1 hms1 hmt1 hnm1 hnt1 hom2 hom3 hom6 hop2 hos1 hos3 hrk1 hrt3 hsf1 hsl7 hsp12 hsp150

hsp31 htd2 hts1 hua2 hul4 hul5 hvg1 hxk1 hxk2 hxt10 hxt5 hxt6 hym1 idp2 ids2 ilv3 ilv5 ilv6 ime1 ime2 imh1 iml2 imp1 imp2' inh1 inm1 ino2 inp2 inp53 inp54 ipt1 iqg1 ira1 ire1 isa1 isa2 ist2 itr2 ivy1 ixr1 izh3 jhd1 jid1 jip4 jip5 jjj2 kap120 kcs1 kel2 kex1 kic1 kin3 kin4 kip2 kip3 kkq8 kns1 kre2 kre5 kre6 krs1 ktr3 ktr4 ktr5 ktr6 laa1 lag2 lap3 las1 lcb4 lcb5 ldb19 ldb7 lem3 leu1 leu2 leu3 lhs1 lia1 lif1 lin1 lip2 lip5 los1 lot6 lpe10 lro1 lsb1 lsb5 lsc1 lsc2 lst4 ltp1 lys1 lys2 lys21 lys5 lys9 mac1 mak16 mal12 mal32 map1 map2 mbf1 mca1 mcm4 mcr1 mcx1 mdh2 mdh3 mdj1 mdm1 mdm20 mdm30 mdm31 mdm32 mdr1 mef1 mei5 mep3 mer1 mes1 met16 met22 met6 mga2 mgs1 mhp1 mid1 mig1 mih1 mip6 mks1 mlc2 mmr1 mms1 mms4 mmt2 mnd1 mnn4 mnn5 mnp1 mnr2 mot3 mpc54 mph1 mpt5 mrh1 mrh4 mrm1 mrp2 mrpl11 mrpl31 mrpl32 mrpl37 mrpl38 mrpl44 mrpl49 mrpl50 mrpl51 mrs1 msa2 msb1 msb3 msc2 msc3 msc7 msd1 mse1 msk1 msl5 msm1 msn4 mss1 mss116 mss18 mss4 mst27 mst28 mtg1 mtq1 mtr10 mub1 muc1 mud2 mvd1 nas2 nat3 ncb2 nce102 ncl1 ncp1 ncs2 ndd1 nde1 ndl1 nej1 nem1 nfu1 ngl2 ngr1 nis1 nma1 nma2 npp2 npr2 npt1 nrg1 nrg2 nrp1 nta1 nte1 ntf2 nth1 nth2 num1 nup188 nup192 nus1 nvj1 oac1 oaf3 ogg1 ole1 om45 oms1 opi1 opy1 orm2 osh2 osh3 osh6 osm1 oye3 paa1 pac1 pac2 pad1 pam1 pam17 pan2 pan3 pbp1 pbp2 pca1 pcl1 pcl10 pcl2 pcl8 pcl9 pcm1 pct1 pdc2 pdc5 pdc6 pde2 pdi1 pdr1 pdr10 pdr11 pdr15 pdr17 pdr3 pdr5 pds1 pdx3 pea2 pep1 pep4 pep7 pet10 pet127 pet309 pet9 pex1 pex15 pex19 pex25 pex27 pex3 pex30 pex32 pex6 pfk27 pfs1 pfy1 pga2 pgm1 pgs1 pgu1 pho13 pho80 pho84 pho86 pho88 pho89 pho91 phr1 pib1 pib2 pig2 pih1 pik1 pim1 pin3 pin4 pip2 pkh1 pkh2 pkh3 pkr1 plb3 plc1 pma2 pmd1 pmi40 pml39 pmt4 pnc1 png1 pom152 pom34 pox1 ppa2 ppe1 ppn1 ppq1 pps1 ppt1 ppx1 prk1 prm2 prm8 pro1 pro2 pro3 prp22 prp28 prp5 prs1 prs2 prs4 psd1 psd2 psk2 pso2 psp2 pst1 ptc4 ptc5 ptk2 ptp2 ptp3 puf3 pus1 pus2 pus4 pus7 pus9 pxr1 pyc1 pyc2 pyk2 q0140 qdr2 qns1 qri1 rad14 rad2 rad27 rad28 rad33 rad34 rad54 rad9 rap1 rat1 rbg1 rck2 rcr1 rdi1 rdr1 rds2 reg2 reh1 rei1 rex2 rft1 rga1 rga2 rgd1 rgp1 rgs2 rgt2 rho2 rho4 rhr2 ria1 rib4 ric1 rim101 rim13 rim15 rim4 rio1 ris1 rkm2 rkr1 rlf2 rlm1 rma1 rmd8 rml2 rmt2 rnh1 rnr1 rog1 rog3 rot1 rox1 rph1 rpl14a rpl15b rpl19a rpl21b rpl22a rpl22b rpl24b rpl34a rpl34b rpl35a rpl37a rpl37b rpl39 rpl41a rpl41b rpl42a rpl43a rpl43b rpn4 rpp1a rpp1b rps10a rps12 rps16a rps18b rps19a rps21a rps21b rps23b rps25b rps26a rps27a rps27b rps28b rps29a rps29b rps31 rrf1 rrm3 rrn3 rrp14 rrp3 rsb1 rsf2 rsm28 rsn1 rtg1 rtg3 rtn1 rtn2 rts3 rtt103 rtt105 rtt106 sac1 sac7 sae2 sae3 saf1 sam2 san1 sap1 sap190 sap4 sbe22 sch9 scj1 scm3 scp1 scw4 sdp1 sds24 sec11 sec18 sed4 sef1 sen2 sen34 seo1 ser1 set4 sfa1 sfk1 sfl1 sfp1 sga1 sgd1 sge1 sgm1 she4 shm1 shq1 shr5 sip4 sis1 skg3 skm1 skn1 skn7 sko1 sla2 slf1 slk19 slm3 sln1 slo1 slp1 slx1 slx8 smf3 smp1 smy2 sna3 sna4 snl1 sno2 sno4 snq2 snx4 snx41 sod2 sog2 sok1 som1 sop4 spc3 spe2 spe3 spe4 spf1 spo13 spo20 spo7 spo77 spp2 spt14 sqs1 sqt1 srl3 sro77 srp40 sry1 sse2 ssf2 ssh1 ssh4 ssl2 ssp2 sst2 ssy5 stb1 stb3 stb5 ste23 stf1 stn1 stp1 stp3 stp4 sts1 stt4 stu1 sub1 sur4 svl3 svp26 swa2 swe1 syg1 syp1 sys1 tad2 tad3 tah1 tah11 tah18 tam41 tan1 taz1 tbs1 tcb1 tcb2 tcm62 tdp1 ted1 tel1 ten1 tfa2 tgl1 thg1 thi21 thi22 thi3 thi72 tho1 thr1 thr4 ths1 tif11 tif3 tim11 tim13 tim8 tip1 tip41 tir1 tir4 tis11 tkl1 tkl2 tma108 tma20 tma46 tma64 tok1 tos1 tos3 tos4 tpa1 tpm1 tpt1 tre1 tri1 trk1 trm1 trm12 trm3 trm5 trm8 trm82 trp2 trp5 tsa2 tsc10 tvp15 tvp18 tye7 tyr1 tys1 tyw1 uba2 uba4 ubc1 ubc6 ubc8 ubp1 ubp12 ubp13 ubp9 ubr1 ubr2 ubx3 ubx4 ubx5 ubx6 ubx7 upc2 ura1 ura6 ura8 ure2 urm1 uso1 uth1 utp11 utp14 utp23 utr2 vac14 vac17 vac7 van1 vas1 vhr1 vhs1 vmr1 vps64 vps73 vps74 vps9 vrp1 vth2 vts1 war1 whi3 wrs1 xks1 xrs2 yal046c yap1801 yap7 yar009c yar062w ybl005w-a ybl005w-b ybl010c ybl029w ybl036c ybl044w ybl054w ybl055c ybl059w ybl081w ybl086c ybl104c ybl111c ybp1 ybp2 ybr028c ybr030w ybr047w ybr062c ybr063c ybr071w ybr108w ybr139w ybr184w ybr194w ybr238c ybr242w ybr259w ybr287w yck2 yck3 ycl019w ycl042w ycl045c ycl047c ycr016w ycr076c ycr087c-a ycr090c ycr102c yct1 ydl063c ydl085c-a ydl086w ydl124w ydl144c ydl156w ydl199c ydl203c ydl241w ydr034c-d ydr049w ydr051c ydr115w ydr131c ydr170w-a ydr179w-a ydr186c ydr196c ydr210w-b ydr239c ydr248c ydr261w-b ydr266c ydr291w ydr306c ydr326c ydr341c ydr348c ydr381c-a ydr412w ydr415c ydr520c ydr545w yel001c yel007w yel025c yel047c yel057c yel070w yel1 yen1 yer004w yer067w yer077c yer078c yer089c yer138c yer139c yer140w yer158c yer160c

yer182w yer184c yfl002w-a yfl042c yfl046w yfl054c yfr006w yfr011c yfr043c ygk3 ygl039w ygl057c ygl081w ygl082w ygl108c ygl117w ygl140c ygl146c ygl157w ygp1 ygr012w ygr017w ygr052w ygr066c ygr067c ygr068c ygr093w ygr102c ygr111w ygr126w ygr130c ygr150c ygr161c-c ygr161w-b ygr205w ygr207c ygr210c ygr237c ygr266w ygr277c ygr296w yhl008c yhl021c yhl039w yhm2 yhp1 yhr001w yhr035w yhr080c yhr097c yhr112c yhr127w yhr131c yhr138c yhr182w yhr199c yhr202w yih1 yil055c yil067c yil077c yil096c yil108w yil127c yil151c yil177c yip3 yir007w yir024c yjl016w yjl045w yjl046w yjl051w yjl062w-a yjl070c yjl123c yjl131c yjl132w yjl144w yjl147c yjl149w yjl206c yjr024c yjr026w yjr027w yjr028w yjr029w yjr061w yjr096w yjr098c yjr100c yjr136c yjr141w yjr156c ykl027w ykl037w ykl047w ykl050c ykl069w ykl151c ykl161c ykl215c ykr011c ykr015c ykr018c ykr023w ykr043c ykr051w ykr070w ykr096w ylf2 yll029w yll032c yll054c ylr003c ylr035c-a ylr057w ylr064w ylr072w ylr132c ylr149c ylr152c ylr157c-a ylr194c ylr211c ylr218c ylr227w-a ylr241w ylr243w ylr256w-a ylr267w ylr271w ylr278c ylr287c ylr290c ylr312c ylr326w ylr352w ylr356w ylr363w-a ylr392c ylr407w ylr410w-b ylr419w ylr422w ylr426w yme2 yml002w yml020w yml030w yml040w ymr010w ymr034c ymr045c ymr046c ymr050c ymr086w ymr098c ymr102c ymr111c ymr124w ymr134w ymr144w ymr155w ymr181c ymr187c ymr196w ymr209c ymr226c ymr315w ynd1 ynl022c ynl040w ynl045w ynl050c ynl054w-b ynl092w ynl122c ynl134c ynl155w ynl157w ynl168c ynl176c ynl181w ynl187w ynl208w ynl213c ynl234w ynl247w ynl254c ynl284c-a ynl284c-b ynl311c ynr014w ynr021w ynr029c ynr062c ynr065c yol022c yol057w yol070c yol103w-a yol154w yop1 yor021c yor051c yor052c yor059c yor093c yor097c yor164c yor192c-c yor205c yor215c yor220w yor227w yor238w yor251c yor262w yor287c yor289w yor304c-a yor305w yor342c yor356w yor388c ypd1 ypi1 ypk1 ypl009c ypl014w ypl099c ypl105c ypl113c ypl150w ypl158c ypl183w-a ypl191c ypl199c ypl206c ypl222w ypl225w ypl236c ypl247c ypl257w-b ypp1 ypr003c ypr015c ypr045c ypr078c ypr081c ypr083w ypr084w ypr085c ypr089w ypr114w ypr115w ypr152c ypr174c yps7 ypt10 ypt32 ypt35 yrr1 ysp1 ysw1 yta6 yvh1 zap1 zeo1 zim17 zpr1 zrc1 zrg17 zrg8

Cluster 1, 88 Proteins

aar2 brr1 brr2 bud13 bud31 cbc2 cdc40 cef1 clf1 cus1 cwc15 cwc2 cwc21 cwc22 cwc23 cwc25 dcp1 dcp2 dhh1 dib1 ecm2 edc1 edc3 gcn2 hsh155 hsh49 ist3 isy1 lea1 lsm1 lsm2 lsm3 lsm4 lsm5 lsm6 lsm7 lsm8 luc7 msl1 mud1 nam8 ntc20 ntr2 pat1 pby1 pml1 prp11 prp16 prp19 prp2 prp21 prp24 prp3 prp31 prp38 prp39 prp4 prp40 prp42 prp45 prp46 prp6 prp8 prp9 rds3 rse1 slu7 smb1 smd1 smd2 smd3 sme1 smx2 smx3 snp1 snt309 snu114 snu23 snu56 snu66 snu71 spp381 spp382 sto1 syf1 syf2 yhc1 yju2

Cluster 2, 288 Proteins

abd1 act1 ada2 apd1 arp4 arp5 arp6 arp8 ash1 asm4 bdf1 bfa1 bre2 bub2 bud27 cdc31 cdc73 cft1 cft2 chd1 chl1 cka1 cka2 ckb1 ckb2 clp1 cnm67 cse2 csl4 cti6 ctr9 dep1 dis3 dst1 dyn2 eaf1 eaf3 eaf5 eaf6 eaf7 elp2 elp3 elp4 elp6 epl1 esa1 fip1 gal11 gal4 gap1 gcn4 gcn5 gim3 gim4 gim5 gis1 gle2 hca4 hfi1 hot1 htz1 ies1 ies2 ies3 ies4 ies5 ies6 iki1 iki3 ino80 ism1 iwr1 kap114 kar1 kti12 leo1 lrp1 maf1 med1 med11 med2 med4 med6 med7 med8 mex67 mht1 mlp1 mlp2 mob1 mob2 mpe1 mps1 mtr2 mtr3 nab2 ngg1 nhp10 nic96 nsp1 nto1 nud1 nup1 nup100 nup116 nup120 nup133 nup145 nup157 nup159 nup170 nup2 nup49 nup53 nup57 nup60 nup82 nup84 nup85 nut1 nut2 pac10 paf1 pap1 pcf11 pfd1 pfs2 pgd1 pho23 pob3 pta1 pti1 rco1 ref2 ret1 rgr1 rna14 rna15 rox3 rpa12 rpa135 rpa14 rpa190 rpa34 rpa43 rpa49 rpb10 rpb11 rpb2 rpb3 rpb4 rpb5 rpb7 rpb8 rpb9 rpc10 rpc11 rpc17 rpc19 rpc25 rpc31 rpc34 rpc37 rpc40 rpc53 rpc82 rpd3 rpo21 rpo26 rpo31 rrp4 rrp40 rrp42 rrp43 rrp45 rrp46 rrp6 rrp8 rtf1 rvb1 rvb2 rxt2 rxt3 sac3 sap30 sas3 sdc1 sds3 sec13 sec16 sec23 sec24 sec31 set1 set2 sfb2 sgf11 sgf29 sgf73 shg1 shr3 sin3 sin4 ski6 ski7 soh1 spc110 spc29 spc42 spc72 spc97 spc98 spn1 spp1 spt15 spt16 spt20 spt3 spt4 spt5 spt6 spt7 spt8 srb2 srb4 srb5 srb6 srb7 srb8 srm1 ssn2 ssn3 ssn8 ssu72 swc3 swc4 swc5 swc7 swd1 swd2 swd3 swr1 syc1 taf1 taf10 taf11 taf12 taf13 taf14 taf2 taf3 taf4 taf5 taf6 taf7 taf8 taf9 tfc1 tfc3 tfc4 tfc6 tfc7 tfc8 tfg1 tfg2 thp1 tra1 tub4 ubp8 ulp1 ume1 ume6 vps71 vps72 yaf9 yap1 ybr111w-a ydr026c ygl100w yhi9 yke2 yll023c yng1 yng2 ynr024w ysh1 yta7 yth1

Cluster 3, 4 Proteins
acb1 fre7 q0110 ste2
Cluster 4, 6 Proteins
acp1 atp1 atp11 atp12 fmc1 isd11
Cluster 5, 56 Proteins
add66 blm10 cin8 ecm29 hsm3 nas6 pba1 pol4 ppg1 pph21 pph22 pre1 pre10 pre2 pre3 pre4 pre5 pre6 pre7 pre8 pre9 pup1 pup2 pup3 rad23 rpn1 rpn10 rpn11 rpn12 rpn13 rpn14 rpn2 rpn3 rpn5 rpn6 rpn7 rpn8 rpn9 rpt1 rpt2 rpt3 rpt4 rpt5 rpt6 rrd1 rrd2 rts1 scl1 sem1 sgo1 spg5 tap42 tos8 tpd3 ubp6 ump1
Cluster 6, 5 Proteins
ade16 ade17 sol1 sol2 ylr257w
Cluster 7, 222 Proteins
afg2 alb1 arx1 bfr2 bms1 brx1 bud20 bud21 cbf5 cic1 dbp10 dbp7 dbp9 dim1 dip2 drs1 dss4 ebp2 ecm16 emg1 enp1 enp2 erb1 esf1 esf2 faf1 fpr4 fun12 gbp2 has1 hcr1 hpr1 hrb1 imp3 imp4 ipi1 ipi3 kem1 kre33 kri1 krr1 lcp5 loc1 ltv1 mak11 mak21 mak5 mdn1 mft1 mpp10 mrt4 naf1 nan1 nip1 nip7 nob1 noc2 noc3 noc4 nog1 nog2 nop1 nop12 nop13 nop14 nop15 nop16 nop2 nop4 nop58 nop7 nop9 nsa1 nsa2 nug1 pno1 pol5 prt1 puf4 puf6 pwp2 rai1 rcl1 rio2 rix1 rix7 rli1 rlp24 rlp7 rlr1 rok1 rpf1 rpf2 rpg1 rpl10 rpl11b rpl12a rpl12b rpl13a rpl13b rpl15a rpl16a rpl16b rpl17a rpl17b rpl18a rpl18b rpl19b rpl1a rpl1b rpl20a rpl20b rpl21a rpl24a rpl25 rpl26a rpl26b rpl27a rpl27b rpl28 rpl29 rpl2a rpl2b rpl3 rpl30 rpl31a rpl31b rpl32 rpl33a rpl33b rpl35b rpl36a rpl38 rpl42b rpl4a rpl5 rpl6a rpl6b rpl7a rpl7b rpl8a rpl8b rpl9a rpp0 rpp2b rps10b rps11a rps11b rps13 rps14a rps14b rps15 rps16b rps17b rps18a rps19b rps1a rps1b rps2 rps20 rps22a rps22b rps23a rps24a rps24b rps3 rps30a rps4a rps4b rps5 rps6a rps6b rps7a rps7b rps8a rps9a rps9b rrp1 rrp12 rrp15 rrp5 rrp9 rrs1 rsa4 sbp1 sda1 sik1 sof1 spb1 spb4 sro9 ssf1 sub2 swt1 tex1 thp2 tif34 tif35 tif5 tif6 tsr1 urb1 utp10 utp13 utp15 utp18 utp20 utp21 utp22 utp30 utp4 utp5 utp6 utp7 utp8 utp9 yar1 ydr012w yer102w yhl001w ypl249c-a ytm1
Cluster 8, 35 Proteins
afg3 anp1 atg16 atg5 fet3 hoc1 mnn10 mnn11 mnn9 ost1 ost2 ost3 ost4 ost5 ost6 phb1 phb2 pkc1 pmr1 pmu1 rer1 sbh1 sbh2 sec61 sec62 sec63 sec66 sec72 spc2 sss1 stt3 swp1 wbp1 yer064c yta12
Cluster 9, 13 Proteins
air1 air2 ccp1 cdc33 mtr4 nab3 nab6 nrd1 pap2 sen1 tif4631 tif4632 trf5
Cluster 10, 4 Proteins
ald2 ald3 ykl100c ylr143w
Cluster 11, 3 Proteins
alg1 alg11 alg2
Cluster 12, 5 Proteins
aly2 apl1 apl3 apm4 aps2
Cluster 13, 25 Proteins
ame1 chl4 cse4 ctf19 ctf3 dsn1 iml3 mcm16 mcm21 mcm22 mif2 mtw1 nkp1 nkp2 nnf1 nsl1 nuf2 okp1 prp18 spc105 spc24 spc25 tid3 ydl073w ydr532c
Cluster 14, 3 Proteins
ams1 atg19 lap4
Cluster 15, 13 Proteins
apc1 apc11 apc2 apc4 apc5 apc9 cdc16 cdc23 cdc26 cdc27 doc1 mnd2 swm1
Cluster 16, 5 Proteins
apl2 apl4 apm2 aps1 yfl034w
Cluster 17, 4 Proteins

apl5 apl6 apm3 aps3

Cluster 18, 10 Proteins

arc15 arc18 arc19 arc35 arc40 arp2 arp3 myo3 myo5 ygl242c

Cluster 19, 14 Proteins

arf1 arf2 bch1 bch2 bud7 bug1 chs3 chs5 chs6 gga1 gpi16 grh1 pfa4 yhr098c

Cluster 20, 4 Proteins

arg80 arg81 mcm1 yox1

Cluster 21, 4 Proteins

arp1 jnm1 nip100 ydr106w

Cluster 22, 40 Proteins

abf2 arp7 arp9 dls1 dpb4 hap4 htl1 ioc2 ioc3 ioc4 isw1 isw2 itc1 mot1 nfi1 nhp6b npl6 reb1 rfx1 rsc1 rsc2 rsc3 rsc30 rsc4 rsc58 rsc6 rsc8 rsc9 rtt102 sfh1 snf11 snf12 snf2 snf5 snf6 sth1 swi1 swi3 swp82 vps1

Cluster 23, 4 Proteins

asi1 asi3 yae1 ynl260c

Cluster 24, 12 Proteins

ask1 dad1 dad2 dad4 dam1 duo1 hsk3 nca2 npy1 spc19 spc34 ybr233w-a

Cluster 25, 7 Proteins

atg1 atg13 atg17 atg29 cis1 ubp11 ybr197c

ClusterCluster 26, 5 Proteins

atg14 vps15 vps30 vps34 vps38

Cluster 27, 4 Proteins

atg18 atg2 atg23 atg9

Cluster 28, 4 Proteins

atg3 atg7 atg8 atp14

Cluster 29, 6 Proteins

atp10 atp4 atp6 atp8 oli1 oxa1

Cluster 30, 13 Proteins

avo1 avo2 bit61 kog1 lst8 slm1 slm2 tco89 tor1 tor2 tsc11 yfr039c ylr454w

Cluster 31, 4 Proteins

axl1 axl2 bud5 erv14

Cluster 32, 4 Proteins

bbp1 mps2 mps3 nbp1

Cluster 33, 5 Proteins

bcp1 rkm1 rpl23a rpl23b ymr1

Cluster 34, 46 Proteins

bcs1 coa1 cob cor1 cox1 cox14 cox15 cox4 cox5a cox6 cox9 cyt1 mdm10 mdm12 mge1 mgr1 mmm1 mpm1 mrs11 mrs5 mss51 pam16 pam18 qcr2 qcr6 rip1 sam35 sam37 sam50 shy1 ssq1 tim17 tim18 tim21 tim22 tim23 tim44 tim50 tim54 tim9 tom20 tom22 tom40 tom5 tom70 yme1

Cluster 35, 10 Proteins

bcy1 jjj1 mrs6 tpk1 tpk2 tpk3 vps21 ymr258c ypt52 ypt53

Cluster 36, 8 Proteins

bem1 cdc24 cdc42 fus3 rsr1 ste11 ste5 ste7

Cluster 37, 74 Proteins

akr1 bet1 bos1 btn2 ccz1 cog1 cog2 cog3 cog4 cog5 cog6 cog7 cog8 cop1 ddi1 dsl1 egd1 erv41 erv46 glo3 gos1 gpt2 gtb1 ho mon1 mso1 nyv1 pep12 pep3 pep5 rcn1 ret2 ret3 rot2 sec1 sec17 sec20 sec21 sec22 sec26 sec28 sec39 sec9 sed5 sft1 sly1 snc1 snc2 sro7 sso1 sso2 syn8 tip20 tlg1 tlg2 ufe1 ufo1 use1 vam3 vam6 vam7 vps16 vps3 vps33 vps41 vps45 vps51 vps52 vps53 vps54 vps8 vti1 ykt6 ypt7

Cluster 38, 12 Proteins

bet3 bet5 gsg1 gyp6 kre11 nhx1 trs120 trs130 trs20 trs23 trs31 trs33

Cluster 39, 4 Proteins

bik1 bim1 kar9 stu2

Cluster 40, 4 Proteins

bre1 lge1 yhr149c yor365c

Cluster 41, 6 Proteins

brn1 his2 smc2 smc4 ycg1 ycs4

Cluster 42, 11 Proteins

btt1 caf130 caf40 ccr4 cdc36 cdc39 mot2 not3 not5 pop2 yjr011c

Cluster 43, 5 Proteins

bub1 bub3 mad1 mad2 mad3

Cluster 44, 4 Proteins

bud22 gis2 ygr271c-a ygr272c

Cluster 45, 4 Proteins

bud8 bud9 rax1 rax2

Cluster 46, 8 Proteins

cac2 crc1 fet5 fth1 msi1 npr1 rub1 rud3

Cluster 47, 4 Proteins

cam1 efb1 lys4 tef2

Cluster 48, 4 Proteins

cap1 cap2 twf1 yfr016c

Cluster 49, 7 Proteins

cat5 esc8 ime4 msg5 tal1 ygr043c yjl068c

Cluster 50, 8 Proteins

ccl1 kin28 ssl1 tfb1 tfb2 tfb3 tfb4 tfb5

Cluster 51, 6 Proteins

cct2 cct4 cct6 cct7 plp2 vid27

Cluster 52, 8 Proteins

bud4 cdc10 cdc11 cdc12 cdc3 gin4 kcc4 shs1

Cluster 53, 22 Proteins

cdc45 dpb11 erv2 mcm10 mcm2 mcm3 mcm5 mcm6 mcm7 mrc1 pol1 pol12 pri1 pri2 psf1 psf2 psf3 sld3 sld5 srp101 srp102 tof1

Cluster 54, 23 Proteins

cdc48 cue1 der1 doa1 hmg2 hrd1 hrd3 mkc7 npl4 nsg1 otu1 prc1 rim21 shp1 sia1 spt23 ssm4 ubc7 ubx2 ufd1 ufd2 usa1 yos9

Cluster 55, 4 Proteins

cdc50 dnf2 drs2 rcy1

Cluster 56, 4 Proteins

cdc34 cdc4 cdc53 skp1

Cluster 57, 15 Proteins

aqr1 cdc9 ctf18 ctf8 dcc1 eco1 met12 met13 rad24 rfc1 rfc2 rfc3 rfc4 rfc5 yor378w

Cluster 58, 3 Proteins

ceg1 cet1 gdh1

Cluster 59, 3 Proteins

cgi121 gon7 kae1

Cluster 60, 11 Proteins

chc1 clc1 ent3 ent5 gga2 ski2 ski3 ski8 vps27 yap1802 ykl023w

Cluster 61, 4 Proteins

cia1 mfb1 nar1 yhr122w

Cluster 62, 3 Proteins

cin5 tma22 ylr247c

Cluster 63, 5 Proteins

cna1 cnb1 mch1 say1 sfg1

Cluster 64, 5 Proteins

coq3 coq4 coq5 coq6 coq9

Cluster 65, 9 Proteins

cox18 cox2 cox3 mck1 mss2 pnt1 prd1 sco1 sco2

Cluster 66, 4 Proteins

aur1 csg2 csh1 sur1

Cluster 67, 6 Proteins

csi1 csn9 fcp1 pci8 rri1 rri2

Cluster 68, 6 Proteins

csm1 fob1 lrs4 src1 tof2 ydl089w

Cluster 69, 7 Proteins

cst9 msh4 msh5 rad55 rad57 zip1 zip2

Cluster 70, 3 Proteins

cep3 ctf13 sgt1

Cluster 71, 3 Proteins

ctk1 ctk2 ctk3

Cluster 72, 5 Proteins

cup5 ppa1 tfp3 vma21 vma9

Cluster 73, 10 Proteins

cym1 kgd1 kgd2 lat1 lpd1 pda1 pdb1 pdx1 pkp1 ymr31

Cluster 74, 6 Proteins

dap1 glg1 glg2 gsy1 gsy2 guf1

Cluster 75, 7 Proteins

dbp6 leu4 leu9 nop8 rsa3 urb2 yra2

Cluster 76, 5 Proteins

dcs1 dcs2 icl1 trp4 ypl245w

Cluster 77, 7 Proteins

ddc1 mec3 rad17 rev1 rev3 rev7 suv3

Cluster 78, 4 Proteins

ddr48 idp1 ubp3 ymr118c

Cluster 79, 3 Proteins
dhr2 ubp10 ygr251w
Cluster 80, 14 Proteins
adh7 cps1 did4 hcm1 mvb12 snf7 snf8 srn2 stp22 vps20 vps24 vps25 vps28 vps36
Cluster 81, 6 Proteins
dig1 dig2 flo8 mss11 ste12 tec1
Cluster 82, 8 Proteins
ald5 dna2 fen1 msh3 msh6 rad52 rfa2 rfa3
Cluster 83, 3 Proteins
dnm1 fis1 mdv1
Cluster 84, 3 Proteins
avt1 doa4 yef1
Cluster 85, 3 Proteins
dph1 dph2 mnn1
Cluster 86, 4 Proteins
dsk2 lyp1 pdh1 pth2
Cluster 87, 4 Proteins
dug1 dug2 dug3 vth1
Cluster 88, 3 Proteins
dus3 his7 yir035c
Cluster 89, 64 Proteins
ehd3 img1 img2 mam33 mdm38 mhr1 mrp1 mrp10 mrp13 mrp17 mrp20 mrp21 mrp4 mrp49 mrp51 mrp7 mrpl1 mrpl10 mrpl13 mrpl15 mrpl16 mrpl17 mrpl19 mrpl20 mrpl23 mrpl24 mrpl25 mrpl27 mrpl28 mrpl3 mrpl35 mrpl36 mrpl39 mrpl4 mrpl40 mrpl6 mrpl7 mrpl8 mrpl9 mrps16 mrps17 mrps18 mrps28 mrps35 mrps5 mrps8 mrps9 nam9 pet123 rsm10 rsm18 rsm19 rsm22 rsm23 rsm24 rsm25 rsm27 rsm7 sws2 ydr124w yjr101w ylh47 yml6 ynr036c
Cluster 90, 4 Proteins
cul3 elc1 pcl6 rad7
Cluster 91, 5 Proteins
emp24 erp1 erp3 erp5 erv25
Cluster 92, 5 Proteins
end3 gts1 pan1 scd5 sfi1
Cluster 93, 9 Proteins
adh1 eno2 far11 fba1 gpm1 pdc1 pgk1 tpi1 ydr161w
Cluster 94, 3 Proteins
erg10 hsv2 sks1
Cluster 95, 7 Proteins
erg11 erg2 erg25 erg27 erg28 erg3 erg7
Cluster 96, 5 Proteins
est2 est3 pac11 prx1 yrm1
Cluster 97, 7 Proteins
exo70 exo84 sec15 sec3 sec5 sec6 sec8
Cluster 98, 3 Proteins
faa1 fat1 nma111

Cluster 99, 4 Proteins
crf1 fhl1 ifh1 rrp7
Cluster 100, 4 Proteins
cyk3 fox2 mls1 pex11
Cluster 101, 9 Proteins
fyv10 gid7 gid8 moh1 rmd5 vid24 vid28 vid30 ydl176w
Cluster 102, 3 Proteins
fzo1 mgm1 ugo1
Cluster 103, 8 Proteins
gal83 rad30 reg1 sak1 sip1 sip2 snf1 snf4
Cluster 104, 6 Proteins
dbp3 gar1 nhp2 ygr283c yhr072w-a ymr310c
Cluster 105, 10 Proteins
gcd1 gcd11 gcd2 gcd6 gcd7 gcn3 ist1 mrf1 sui2 sui3
Cluster 106, 3 Proteins
cpr3 gdb1 yfr017c
Cluster 107, 8 Proteins
gea1 gmh1 mtq2 pcl7 trm11 trm112 trm9 tyw3
Cluster 108, 4 Proteins
get1 get2 get3 grx7
Cluster 109, 6 Proteins
gir2 prs5 rbg2 yer071c yil152w yir003w
Cluster 110, 4 Proteins
gln4 pbi2 ynl019c yor352w
Cluster 111, 3 Proteins
gor1 nft1 pet122
Cluster 112, 3 Proteins
gab1 gpi17 ptm1
Cluster 113, 4 Proteins
gpi19 gpi2 ras1 ras2
Cluster 114, 4 Proteins
fcf1 gre3 pch2 pmc1
Cluster 115, 4 Proteins
aft1 grx3 grx4 ygl220w
Cluster 116, 6 Proteins
gsp1 gsp2 mog1 rna1 yrb1 yrb30
Cluster 117, 6 Proteins
gtr1 gtr2 meh1 slm4 ycr015c ygr203w
Cluster 118, 3 Proteins
gsf2 hap3 hap5
Cluster 119, 13 Proteins
dbf4 hat1 hat2 hif1 mef2 orc1 orc2 orc3 orc4 orc5 orc6 sir1 yjr154w
Cluster 120, 5 Proteins
hda1 hda2 hda3 msh2 oct1

Cluster 121, 4 Proteins
hhf1 hhf2 hht1 hht2
Cluster 122, 4 Proteins
hir1 hir2 hir3 hpc2
Cluster 123, 11 Proteins
cpr1 hos2 hos4 hst1 rfm1 set3 sif2 snt1 sum1 zds1 zds2
Cluster 124, 4 Proteins
hse1 hua1 rup1 ubp2
Cluster 125, 4 Proteins
hta1 hta2 htb1 htb2
Cluster 126, 3 Proteins
hbt1 hub1 sph1
Cluster 127, 3 Proteins
gln1 hyr1 mxr1
Cluster 128, 11 Proteins
dot6 fpr2 ibd2 mpd2 ndt80 nst1 ppr1 ubp7 ykl075c yol159c-a ypr117w
Cluster 129, 3 Proteins
inp51 irs4 tax4
Cluster 130, 3 Proteins
ipl1 sli15 yer156c
Cluster 131, 3 Proteins
gpb1 gpb2 ira2
Cluster 132, 5 Proteins
cdc5 irr1 scc2 smc1 smc3
Cluster 133, 6 Proteins
isu1 nfs1 sdh1 sdh2 yfh1 ypr004c
Cluster 134, 4 Proteins
jhd2 sas10 ugp1 yil091c
Cluster 135, 3 Proteins
cik1 kar3 vik1
Cluster 136, 3 Proteins
dyn1 kel3 yro2
Cluster 137, 3 Proteins
kex2 sil1 ybt1
Cluster 138, 3 Proteins
lac1 lag1 lip1
Cluster 139, 7 Proteins
crm1 gea2 lcb1 lcb2 mir1 tsc3 yhr020w
Cluster 140, 7 Proteins
lhp1 sec65 srp14 srp21 srp54 srp68 srp72
Cluster 141, 4 Proteins
mae1 psa1 sam1 tub3
Cluster 142, 3 Proteins
mak10 mak3 mak31

Cluster 143, 4 Proteins
dph5 mas1 mas2 msh1
Cluster 144, 5 Proteins
mbp1 nrm1 swi4 swi6 whi5
Cluster 145, 5 Proteins
mbr1 mnl1 nde2 pig1 yjl118w
Cluster 146, 3 Proteins
cdc25 mds3 ybr225w
Cluster 147, 3 Proteins
mdy2 sgt2 ybr137w
Cluster 148, 3 Proteins
mei4 rec107 rec114
Cluster 149, 4 Proteins
hop1 mek1 red1 ymr323w
Cluster 150, 4 Proteins
met28 met30 met31 met4
Cluster 151, 9 Proteins
cox17 cox19 mia40 muk1 pep8 vps17 vps29 vps35 vps5
Cluster 152, 9 Proteins
isn1 mlh1 mlh2 mlh3 pms1 rmi1 sgs1 tep1 top3
Cluster 153, 3 Proteins
mmf1 ydr018c ydr374c
Cluster 154, 8 Proteins
met17 mms2 rad18 rad5 sip5 siz1 ubc13 ubc9
Cluster 155, 8 Proteins
kre29 mms21 nse1 nse3 nse4 nse5 smc5 smc6
Cluster 156, 3 Proteins
dop1 mon2 neo1
Cluster 157, 3 Proteins
abz1 mpa43 tsa1
Cluster 158, 5 Proteins
his6 mrp8 rpl9b yel043w ylr179c
Cluster 159, 3 Proteins
mrs2 sue1 yil169c
Cluster 160, 3 Proteins
msf1 tma16 ynl024c
Cluster 161, 9 Proteins
csr2 kip1 msn2 psr1 psr2 put3 sok2 vhs2 whi2
Cluster 162, 4 Proteins
ecm27 mtf1 pzf1 rpo41
Cluster 163, 9 Proteins
mtf2 sls1 urh1 vab2 ydr357c ygl079w ykl061w ylr408c ynl086w
Cluster 164, 4 Proteins
mum2 sma1 spo14 ygl036w

Cluster 165, 4 Proteins
myo4 she2 she3 sul2
Cluster 166, 5 Proteins
bye1 nam2 rif1 rif2 ydl173w
Cluster 167, 3 Proteins
ard1 nat5 rim8
Cluster 168, 3 Proteins
cfd1 nbp35 tao3
Cluster 169, 3 Proteins
cup9 ndc1 ypt11
Cluster 170, 4 Proteins
cos3 nha1 yhl010c ykr017c
Cluster 171, 4 Proteins
lys14 nmd2 sur7 upf3
Cluster 172, 3 Proteins
nuc1 trz1 ymr099c
Cluster 173, 7 Proteins
oca1 oca2 rec8 siw14 ycr095c ydr067c yhl029c
Cluster 174, 4 Proteins
idp3 pet111 pet494 tfs1
Cluster 175, 7 Proteins
pet130 rpm2 ydr065w yhr054c yll033w ylr091w ypr116w
Cluster 176, 9 Proteins
mmt1 pex10 pex12 pex13 pex14 pex17 pex2 pex5 pex8
Cluster 177, 3 Proteins
pex18 pex7 pot1
Cluster 178, 4 Proteins
alt1 bir1 pfk26 ygr031w
Cluster 179, 3 Proteins
pho11 pho5 yfr055w
Cluster 180, 3 Proteins
pcl5 pho81 pho85
Cluster 181, 3 Proteins
apn1 cbs1 pir1
Cluster 182, 3 Proteins
pmt2 pmt3 pmt5
Cluster 183, 3 Proteins
cyc7 irc25 poc4
Cluster 184, 3 Proteins
pol3 pol31 pol32
Cluster 185, 11 Proteins
pop1 pop3 pop4 pop5 pop6 pop7 pop8 rmp1 rpp1 rpr2 snm1
Cluster 186, 3 Proteins
pph3 psy2 psy4

Cluster 187, 8 Proteins
mns1 ppz1 ppz2 rdh54 sis2 trm2 vhs3 ykl088w
Cluster 188, 3 Proteins
pst2 rfs1 ycp4
Cluster 189, 3 Proteins
are2 nbp2 ptc1
Cluster 190, 3 Proteins
kes1 pxa1 pxa2
Cluster 191, 3 Proteins
cdc43 ram1 ram2
Cluster 192, 16 Proteins
rav1 rav2 stv1 tfp1 vma10 vma13 vma2 vma4 vma5 vma6 vma7 vma8 vph1 xdj1 yig1 ymr027w
Cluster 193, 4 Proteins
cad1 rck1 rod1 sol4
Cluster 194, 3 Proteins
rec102 rec104 spo11
Cluster 195, 3 Proteins
mth1 rgt1 std1
Cluster 196, 3 Proteins
rnh201 rnh202 rnh203
Cluster 197, 5 Proteins
rrn11 rrn6 rrn7 slm5 sps22
Cluster 198, 4 Proteins
rrn10 rrn5 rrn9 uaf30
Cluster 199, 3 Proteins
sas2 sas4 sas5
Cluster 200, 3 Proteins
ptc7 sec14 ykl091c
Cluster 201, 3 Proteins
mip1 sed1 sit1
Cluster 202, 3 Proteins
ser3 ser33 yhr215w
Cluster 203, 3 Proteins
hkr1 msb2 sho1
Cluster 204, 3 Proteins
clb3 clb4 sic1
Cluster 205, 3 Proteins
snt2 xbp1 ylr413w
Cluster 206, 5 Proteins
fbp26 ftr1 snx3 ste13 ylr345w
Cluster 207, 5 Proteins
plm2 spo75 stb4 yhr198c ykr078w
Cluster 208, 3 Proteins
abf1 srl2 yhl009w-b

Cluster 209, 3 Proteins
ssa1 ssa2 ssb1
Cluster 210, 3 Proteins
ssk1 ssk2 ssk22
Cluster 211, 3 Proteins
gpa1 ste18 ste4
Cluster 212, 3 Proteins
mba1 opy2 ste50
Cluster 213, 3 Proteins
tbf1 vid22 ygr071c
Cluster 214, 3 Proteins
toa1 toa2 yfr032c
Cluster 215, 3 Proteins
tps2 tps3 tsl1
Cluster 216, 5 Proteins
clb1 cue5 trm7 ymr259c ypl183c
Cluster 217, 3 Proteins
rho5 trr1 trr2
Cluster 218, 3 Proteins
ade12 sen54 tuf1
Cluster 219, 3 Proteins
tus1 ycf1 ypl066w
Cluster 220, 3 Proteins
tvp23 yip4 yip5
Cluster 221, 4 Proteins
qcr7 tul1 ubc4 ubc5
Cluster 222, 3 Proteins
uba3 ubc12 ula1
Cluster 223, 3 Proteins
tgl2 ura10 ura5
Cluster 224, 3 Proteins
cmk1 vma22 vph2
Cluster 225, 3 Proteins
fms1 rtt109 vps75
Cluster 226, 3 Proteins
did2 vps60 vta1
Cluster 227, 4 Proteins
vtc1 vtc2 vtc3 vtc4
Cluster 228, 4 Proteins
rnr4 wtm1 wtm2 yor283w
Cluster 229, 6 Proteins
pet112 yal049c ydl025c ygr016w yhr009c ypk2
Cluster 230, 3 Proteins
eki1 suc2 yhr087w

Cluster 231, 5 Proteins
yer074w-a yif1 yip1 ypt1 ypt31
Cluster 232, 4 Proteins
jsn1 rbl2 ydr332w yjl107c
Cluster 233, 4 Proteins
hol1 mmp1 plb1 yjr012c
Cluster 234, 3 Proteins
fol1 ura3 ymr074c
Cluster 235, 3 Proteins
din7 fum1 ymr291w
Cluster 236, 3 Proteins
kin1 ydr287w ynl035c
Cluster 237, 3 Proteins
kin82 ybr235w ynr047w
Cluster 238, 4 Proteins
sag1 trl1 yjr124c ypl141c
Cluster 239, 3 Proteins
svf1 yjl217w ypr118w
Cluster 240, 3 Proteins
lsb3 sla1 ysc84
