# Supplementary material for: A structural approach for finding functional modules from large biological networks
Source: BMC Bioinformatics. 2008 Aug 12;9(Suppl 9):S19. doi: 10.1186/1471-2105-9-S9-S19 (PMC2537570; doi:10.1186/1471-2105-9-S9-S19)
Supplement: Additional File 2 — Clustering result of CNM for PPI network. [file 1471-2105-9-S9-S19-S2.pdf]

Cluster 1,   7 Proteins
rpm2 gds1 ymr034c pet130 ygl157w ypr116w yhr054c
Cluster 2,   25 Proteins
mdy2 aly2 apl1 apl3 pcl7 apm4 aps2 arp1 nip100 ydr106w ubp10 sgt2 gea1 ygr251w trm112 lys9 gmh1 tyw3 ubp12 jnm1 yor164c ybr137w mtq2 trm11 trm9
Cluster 3,   2 Proteins
imp1 som1
Cluster 4,   3 Proteins
ser3 ser33 yhr215w
Cluster 5,   4 Proteins
ubc4 qcr7 ubc5 tul1
Cluster 6,   4 Proteins
spo75 plm2 ykr078w yhr198c
Cluster 7,   4 Proteins
ald2 ald3 ykl100c ylr143w
Cluster 8,   2 Proteins
ynl035c kin1
Cluster 9,   3 Proteins
yil152w yer071c gir2
Cluster 10,   2 Proteins
alg13 alg14
Cluster 11,   107 Proteins
aco2 mrpl16 aep1 mhr1 ymr098c mrps35 mrp7 mrpl19 mam33 ydr124w mrps5 atg26 mrpl9 ehd3 fyv4 mrps16 rsm24 rsm25 mrpl17 mrpl3 mrp1 mrp4 shq1 mrps28 mrpl1 rsm18 mrp13 ypr015c mrpl10 cox11 mrpl36 mrpl24 mrp51 mrps18 mrps9 rsm10 rsm22 rsm23 yjr101w yml6 ynl168c mrp21 mrps17 nam9 rsm7 fig2 mrp2 mrh4 mrpl50 img1 img2 mdm38 mrp49 mrpl13 mrpl15 mrpl20 mrpl23 mrpl27 mrpl28 mrpl35 mrpl4 mrpl6 mrpl7 mrpl8 ylh47 mrp20 mrpl25 mrpl40 inm1 jsn1 yjl107c yjr096w mrp10 pet123 rsm27 rsm28 tho1 ykl151c mrp17 mrpl11 mrpl38 mrpl49 mrpl51 q0140 rml2 sws2 ypl183w-a mrps8 rsm19 yil067c ynr036c mrpl31 mrpl32 mrpl39 mrpl44 mrpl37 ygr093w ydr115w ydr332w tys1 ygr150c ynl022c yor205c mvd1 rbl2 yjr136c ysp1
Cluster 12,   3 Proteins
eki1 yhr087w suc2
Cluster 13,   919 Proteins
rfa3 spt2 kap95 abd1 rpb2 rpo21 spt4 spt5 spt6 abf2 act1 arp9 ase1 chd1 hat1 hhf1 hta2 htb2 isw1 mot1 nhp6a pdr1 rps0b rsc3 rsc6 rsc8 rvb1 sgv1 sin3 slx8 snf5 swi3 top1 vip1 vps1 tup1 yir003w rpc40 pil1 ach1 rfc2 bmh1 mec1 rfa1 rfc4 ola1 arp4 arp5 arp8 cap1 chk1 dld2 eaf5 epl1 esa1 htz1 ies1 ies2 ies3 ino4 ino80 mcm5 myo1 nhp10 nup2 pfy1 rvb2 sec2 srm1 sup35 swc4 swc5 swr1 taf14 vps71 yaf9 yih1 yng2 ada2 adr1 ahc2 cdc31 cdc36 cti6 gal4 gcn4 gcn5 hfi1 hht1 hog1 hta1 med1 med6 med7 ngg1 nut1 sac3 sgf11 sgf29 sgf73 sin4 spt15 spt20 spt3 spt7 spt8 srb2 srb6 taf1 taf10 taf11 taf12 taf3 taf5 taf6 taf9 thp1 tra1 ubp8 yap1 ybr111w-a ade5,7 reb1 adh2 fba1 fun19 isw2 rad16 sir2 air2 fkh2 lcd1 mus81 sld2 pmt1 adh5 adh6 sir3 yer078c sua7 aep3 pkh1 yra1 aft1 cyc8 grx4 hpr1 kap123 not3 rad24 rpa135 ahc1 nrg2 smc1 spt16 cka1 rfa2 mag1 air1 hrb1 mtr4 npl3 pap2 trf5 nab3 nrd1 sen1 srp1 sto1 ald5 rad52 cys4 alg3 med8 rpb9 alg5 scc4 ams1 atg19 ycr043c rpb7 ckb1 pab1 swi1 apn1 hho1 pir1 rim1 apn2 htl1 cst6 gal10 aqr1 ctf18 ara1 pdr3 ara2 lsm12 gus1 mes1 yhr131c ylr247c asr1 pob3 ard1 cka2 hem13 nap1 nat5 snf1 arg80 arg81 mcm1 arg82 psy2 rtt102 ylr278c mcm4 mcm7 san1 arp6 bdf1 eaf1 eaf3 eaf6 eaf7 ies4 ies5 ies6 sap185 swc3 swc7 swi5 vps72 rtg2 skg3 arp7 hap4 nfi1 npl6 psk1 rsc1 rsc2 rsc30 rsc4 rsc58 rsc9 sfh1 snf11 snf12 snf2 snf6 sth1 swp82 ydr520c yil177c ypr081c htb1 srb5 ylr072w ldb7 rpo31 tif4631 tma22 asf1 bdf2 hhf2 hht2 hir1 hir2 hir3 hpc2 qdr2 rad53 rfc1 rfc3 rfc5 rpc19 rtt109 sas2 sas4 sas5 sfp1 taf7 ash1 dep1 pho23 rpd3 rxt2 rxt3 sap30 sds3 ume1 ume6 ask10 bmh2 dst1 ssn8 taf4 rpb11 rpb3 ate1 cdc73 top3 atg11 atg20 atg9 rpa14 atg7 met10 atg23 atg3 mtr10 ris1 ccr4 cdc39 mft1 hat2 yku70 ats1 ceg1 ayt1 paf1 ess1 ckb2 mcd1 bdp1 dop1 yta7 cpr7 ssa3 cdc5 ste23 bfr1 mms22 stb4 yer139c msh3 hsp104 pbp4 sir4 bnr1 csr2 fin1 hap1 mks1 nth1 sfl1 sok1 stu1 svl3 ymr086w ynr047w mig1 pik1 rfx1 ymr031c ynr014w yta6 bop3 rnt1 bpt1 mlp2 bre5 msc7 taf13 yar010c ydr415c brf1 maf1 brl1 dpb3 ylr455w

ymr144w btt1 caf130 yjr011c yml131w bud2 swi6 hmo1 mgm101 ybl044w bud23 bud27 gis1 rpb5 ymr226c bur2 bur6 ncb2 cac2 fet5 pol30 rlf2 rtt106 caf120 caf40 egd2 pop2 ypl216w ssn2 mot2 not5 cap2 dfr1 inp54 tfa1 twf1 cbc2 nab6 tif4632 tir1 urb2 yra2 cbf1 iwr1 scm3 imd3 pol2 rtf1 ssd1 tao3 cbs1 dpb2 ccp1 rnq1 ssn3 tfg2 pph3 ydl156w smc3 mon1 pdi1 top2 pol1 fob1 net1 rpa12 rpa49 tof2 cdc21 ssa1 yro2 clb5 cup2 swi4 yil131c hos3 npy1 ptk2 cse2 gbp2 psp2 cdc9 crt10 pol12 rtt101 ctk1 stp1 ste50 cdc45 mcm2 mcm3 mcm6 mrc1 psf2 sld3 sld5 tof1 doa1 hif1 med4 nut2 ubx4 dbf4 irr1 ivy1 yjr138w orc1 orc5 orc3 ctr9 fol3 gal11 leo1 yor378w cet1 nup116 cfd1 nbp35 taf2 ret1 ctr1 ylr152c eco1 psh1 chz1 gcd14 sgn1 cin5 cit2 pro2 egd1 elf1 hot1 nrg1 pga2 reh1 rim13 rio1 rps10b rrp7 sup45 trl1 vhs3 vps73 ykl088w yor052c sis2 ylr407w fhl1 ifh1 cki1 ssb1 orc6 yil096c orc2 say1 kap114 med2 ypl222w pcl9 cos111 coy1 rpc53 mdh3 gvp36 thp2 crf1 nup159 dot1 rim4 yel001c crp1 med11 pgd1 rgr1 rox3 rpb4 soh1 srb4 srb7 srb8 csl4 dis3 lrp1 mtr3 rrp4 rrp40 rrp42 rrp43 rrp45 rrp46 rrp6 ski6 ski7 yir035c ynr024w csm1 lrs4 src1 ydl089w ygr126w csm3 pdr5 yol154w mcm10 mre11 ctf8 dcc1 ctf4 ctk2 ctk3 sub2 cwp2 hos1 rgt1 set2 sko1 war1 dal81 rpb10 dbp5 gfd1 met12 rrm3 rpc11 rpc17 rpc31 rpc37 rpc82 rpo26 dls1 dpb4 itc1 dna2 fen1 rpa190 dot6 dpb11 psf1 mba1 orc4 sas3 flc2 dsf2 dss1 haa1 tfg1 vth1 rad9 dus3 his7 dyn1 kel3 rco1 nto1 vps75 yjl123c ecm3 ecm5 rpa34 gtb1 sip5 ynl247w msc3 elg1 rad27 elp2 elp3 elp4 elp6 iki1 iki3 kti12 nis1 rsf2 nup1 rrn3 erv2 esc1 exo1 msh2 faa2 snt2 far7 fas2 ymr099c tdh3 ptp3 rck2 rap1 ndd1 fms1 ltp1 rpa43 fps1 frq1 psk2 fyv6 tfa2 gas5 gat1 rlr1 tex1 pde2 mbf1 mlp1 rps29b gcr1 pom152 pom34 gcv3 lip2 ure2 nab2 pri1 taf8 ygr237c gln3 glo4 mth1 grx5 nup60 gtt3 msb3 qri1 uba4 hac1 rim8 hsm3 rpl37a yjr154w phr1 pub1 hfd1 ioc3 mac1 msb1 nuc1 psd2 rrn5 uso1 vth2 yfr006w yng1 jhd1 rph1 ioc4 rad54 rtg3 ycr087c-a mef2 sap4 tri1 pbp2 pkr1 sdo1 ptp2 rpl42a sch9 tok1 pig2 mex67 swt1 ypr089w rfm1 sum1 psy4 rrp8 tbf1 ubc8 vhr1 htd2 yol070c hul4 hxk2 trm82 wrs1 sro77 pus7 rpl9b ime1 imp2' ino2 opi1 ioc2 nhp6b scc2 isa1 nfu1 rpb8 ynl122c ypr174c ogg1 pop3 rpl41a rpl41b rtt103 tan1 kin82 pri2 mse1 ktr6 las1 msh6 leu3 rec8 yjl051w yll032c yhl021c rpc34 mph1 map2 mbp1 nrm1 nde2 ymr046c ynl284c-a ygr012w yox1 tah11 tbs1 ymr102c zrg17 yil077c thi72 met13 mtr2 pso2 mip6 sgs1 ymr187c mms4 mnl1 mnn4 mnr2 mns1 mrs1 spe2 msa2 msc2 msd1 rad2 rpc10 mss4 muc1 trz1 ngl2 pih1 tah1 ydr026c upf3 tif3 rat1 yel047c rim101 ykr015c nrp1 pct1 ymr209c opy2 sir1 thg1 osm1 pcl2 yjl131c stb5 pdr15 pdr17 pex15 rpl34a rpl34b ylr064w plb3 pml39 pmt2 pmt3 ygr266w pmt5 ygr071c stn1 ppn1 psf3 srp101 rpc25 tip41 yor356w ylr413w rdh54 whi5 ras1 zrc1 rnh1 rtt105 yjr141w ycl042w ymr181c rft1 rsb1 ymr1 std1 ria1 yer160c rmi1 rox1 yir024c spn1 ybr259w ypl225w ybl059w yfr011c ydl144c sry1 yfl042c yhr112c seo1 rps31 rrn10 rrn9 uaf30 ybl055c yhr009c rtg1 ybr287w ypl113c yor262w yps7 sip4 trm2 zpr1 snl1 xbp1 yhr035w sts1 srp102 zrg8 ssy5 stb1 ten1 trm12 yjl016w vid22 tir4 trm8 ygr017w ybp1 ybr235w

Cluster 14, 2 Proteins

fig4 kre5

Cluster 15, 220 Proteins

aar2 brr2 prp4 prp8 smb1 smd1 smd2 smx3 snu114 snu66 mrpl22 nas2 acp1 fmc1 isd11 lsm2 nam7 qcr2 apa1 lsm8 ybl104c smd3 atp1 atp12 atp6 atp8 bud31 oli1 atp10 atp11 atp4 oxa1 atp5 pan3 bcs1 cob qcr6 tom22 tom40 snp1 cyk3 ylr345w brr1 mud1 smx2 snu71 cdc40 cef1 clf1 cus1 cwc2 cwc22 cwc23 dib1 hsh155 isy1 lea1 lin1 lsm1 lsm3 lsm4 lsm5 lsm6 lsm7 luc7 pat1 prp16 prp19 prp21 prp3 prp31 prp38 prp39 prp45 prp46 prp9 rse1 sme1 snt309 snu23 spp381 spp382 syf1 ydl085c-a ist2 bud13 kip3 prp22 cat5 coq9 cor1 ime4 prp28 nam8 prp42 snu56 yju2 ynl024c ebs1 slf1 fox2 ntr2 cwc15 cwc21 cwc25 cwc27 msl1 ntc20 prp2 slu7 syf2 yor093c hsh49 nmd2 pby1 cus2 coa1 shy1 coq3 coq4 coq5 coq6 cox14 cox2 cox4 cyt1 fcf1 mih1 rip1 tim17 tim21 tim23 ylr132c cox1 cox3 cox15 cox18 mss2 sco1 sco2 cox5a cox9 cox6 mpm1 csn12 rds3 spp2 cwc24 dcp1 dcp2 edc1 edc3 rlm1 prp24 ssq1 ubp1 fbp26 pmc1 pex11 ftr1 snx3 gre3 pch2 yor097c smp1 mlc2 tep1 inh1 stf1 toa2 yhc1 msf1 prp40 yor238w lot6 ynl092w ygl117w lys14 pam18 mdm10 mdm12 mmm1 sam35 sam37 sam50 tim44 mgr1 yme1 mht1 tim9 mlh3 mls1 mrs11 mrs5 tim18 tim22 tim54 msl5 mud2 mss18 sur7 ycl047c pan2 pam16 tim50 pam17 tom20 tos4 yor287c yhr127w tim13 tom70 tad2 yor305w ste13 tad3 tim8 yfr032c tom5 ydr291w

Cluster 16, 75 Proteins

doc1 mgs1 ama1 cdc16 gtr2 apc1 apc11 apc2 apc4 apc5 cdc23 cdc26 cdc27 mnd2 swm1 apc9 sap1 ygr052w oaf3 yer004w pds5 cca1 azr1 pds1 bio2 mtf1 yjl068c tfb1 ccl1 dcs2 kin28 ssl1 tfb3 tfb4 pol3 ssp2 ygr043c pbp1 cpa1 erg11 tfb2 dcr2 ire1 dcs1 icl1 ypl245w pol31 esc8 ecm27 eps1 erg28 erg2 erg25 erg27 erg7 erg3 erg6 esp1 slk19 gtr1 meh1 slm4 ycr015c ygr203w hal5 itr2 hnt1 pol32 trp4 lys21 tal1 pcm1 mss1 pzf1 rpo41

Cluster 17, 22 Proteins
bas1 pho2 dma1 ydr170w-a ycl019w ydr034c-d ydr210w-b yfl002w-a ygr161w-b yjr026w yjr028w ylr157c-a ylr227w-a ylr256w-a ylr410w-b yml040w yol103w-a ybl005w-a ymr045c ynl284c-b ydr261w-b ygr161c-c
Cluster 18, 2 Proteins
bsd2 tre1
Cluster 19, 3 Proteins
rec102 rec104 spo11
Cluster 20, 11 Proteins
pex14 pex17 pex19 pex10 pex5 mmt1 pex12 pex13 pex2 pex8 pex3
Cluster 21, 6 Proteins
bud5 axl2 erv14 pho11 pho5 yfr055w
Cluster 22, 3 Proteins
mrs2 sue1 yil169c
Cluster 23, 2 Proteins
hop2 mnd1
Cluster 24, 3 Proteins
cyc7 poc4 irc25
Cluster 25, 3 Proteins
yip5 tvp23 yip4
Cluster 26, 3 Proteins
lac1 lag1 lip1
Cluster 27, 3 Proteins
alg1 alg11 alg2
Cluster 28, 3 Proteins
sur1 csg2 csh1
Cluster 29, 3 Proteins
hbt1 hub1 sph1
Cluster 30, 15 Proteins
akl1 hst1 hts1 avt2 sif2 msi1 hos2 hos4 set3 snt1 crc1 tpm1 npr1 ygr296w ymr155w
Cluster 31, 2 Proteins
eri1 spt14
Cluster 32, 2 Proteins
cue3 ykr023w
Cluster 33, 1408 Proteins
aac1 atg12 rad3 skp1 aac3 bud32 hap2 las17 pmr1 rad59 sml1 yck1 aah1 saf1 gcn3 gcn2 prp6 aat2 cdc3 eft2 fun30 sen15 sui1 tdh2 smt3 abp1 app1 ark1 arp2 arp3 crn1 hrr25 hxt7 inp52 lsp1 prk1 rvs161 sac6 scp1 sla1 sps1 srv2 yhr199c ysc84 abz1 mpa43 kns1 ste2 acc1 cct5 dmc1 hfa1 hsp82 mae1 pre1 psa1 shs1 sit4 utp5 vac14 yku80 ace2 clb2 clb3 cln2 fpr1 hxk1 pse1 ubp15 acf2 fir1 lsb1 mlc1 rvs167 tdh1 acf4 gin4 rad26 acm1 cdh1 aco1 ape2 cmd1 dig2 faa4 hsp42 kss1 myo4 sam1 ufd4 yhb1 ypt6 acs2 cdc19 dbp8 eft1 gpm1 hrd1 hsp60 mdn1 rnr1 rpn1 rps0a sam2 sec27 sse1 tef1 tkl1 tub1 tub3 yef3 bem1 bsp1 cyr1 erg13 gcd6 iqg1 myo5 pfk1 sec10 smi1 spo12 ssk2 vma2 add66 arl3 pba1 pre5 ump1 ade1 gcd7 pho4 ste4 ade2 rpn3 ade3 arb1 tma108 uba1 ade4 cys3 lte1 rna1 tpk3 ade6 adh1 adh3 aos1 atg18 bch1 bud7 chs6 ecm1 far11 fth1 gre2 grh1 hsc82 pdc1 pgi1 pgk1 sec7 tpi1 ubp14 yal027w cpr6 hrt3 hym1 bna5 dph1 hsp26 mtf2 sac1 tif2 ybr062c adh4 cdc13 dun1 kap104 bbc1 adk1 cdc14 pho85 prp11 tem1 ubc1 ybl036c ado1 aur1 yil127c cop1 hrt1 ady3 mpc54 pdb1 aep2 swh1 lpd1 afr1 cdc12 msn5 agx1 aha1 aro1 cct4 cdc33 ecm29 gcn1 gfa1 lys12 mgt1 ntg1 slt2 tif1 trp3 tub2 swe1 ahp1 cks1 gpd2 lif1 slx4 ssb2 trx1 trx2 ubc7 urm1 xrs2 yak1 aip1 cof1 ses1 ubp6 rpl40a pep12 ala1 dbf20 tim11 ald4 ald6 idh2 new1 yer089c alf1 alt2 ecm10 nsl1 amn1 ssz1 zuo1 lap4 anb1 hyp2 fks1 mnn1 sec62 uba2 bud4 mdh1 mkt1 ydl241w yer064c ape3 apl2 apl4 apm2 aps1 chc1 chs5 clc1 ent5 gga2 laa1 yfl034w ist1 mad2 eno2 kre6 nma1 sec53 cpr1 fyv8 oye2 pgm2 chs3 cst26 yfr043c apt1 atp3 clu1 fas1 idp1 ilv2 rpg1 rps21b scp160 ssa2 tma46 vma13 ydj1 ydr049w arc1 atg14 cat8 ccc2 cdc10 mca1 ncs2 pet127 pho89 rad10 srs2 ykl050c arc15 arc18 arc19 arc35 arc40 msk1 myo3 sip2 ybr159w nip1 pac1 rad23 ylr241w ynl040w

asc1 eno1 nat1 are2 ptc1 arf1 arf2 bch2 bet1 ctf13 gcs1 gea2 gga1 gpi16 rud3 sec22 sec26 arf3 lsb5 arg1 scl1 arg4 arl1 imh1 mon2 vps53 vps54 slo1 sys1 cct8 gdh1 ksp1 lsb3 rad51 rpn9 rpt1 rpt6 aro2 aro3 ilv5 wtm1 aro4 tma19 ypl150w gnd1 bni1 cbk1 dur1,2 fet3 kap122 met18 mkk2 pan1 pst2 puf3 rnr3 rpn8 rpt3 rpt4 yjr029w mss51 rad1 tap42 shm2 atg16 bem2 mlh2 rkm1 cdc37 nup42 bud14 gpa2 kin3 skt5 ste20 rnr2 rnr4 asm4 nup53 asn1 asn2 ura7 ydr131c atg10 atg5 car2 cpa2 crm1 fet4 gcd11 ipp1 kgd1 ppx1 prb1 ptc7 rex2 rpn10 rpn11 rpn5 rpn6 rpn7 sec18 tcb1 yhr020w yhr033w tfp1 vps15 vps30 vps34 vps38 cdc55 sap155 thi21 vac17 atg4 cdc15 erg20 atp2 def1 atp16 cdc60 ddr48 ynl313c caf4 sui2 yol087c ubc6 atp7 ybl071w-a dpm1 aus1 avo1 avo2 lst8 tor2 tsc11 slm1 slm2 avt4 axl1 ayr1 bar1 bat1 bat2 ynl045w rts3 bck1 ydr326c bck2 bcp1 rpl23a rpl23b ura2 bcy1 cam1 crz1 gdh3 tpk1 tpk2 ypt53 bdh1 bdh2 boi1 boi2 cdc24 cla4 far1 fus3 rga2 ste11 ste5 ste7 kel1 spa2 bem3 yil055c ret2 ret3 sar1 sec21 sec23 sec24 sec28 bfa1 bub2 gpg1 kex2 nud1 cdc7 bgl2 tor1 rpl22a rps29a pbs2 cbf2 bit61 blm10 kkq8 pre10 pre2 pre3 pre4 pre6 pre7 pre8 pre9 pup2 pup3 zds2 kcs1 lcb2 bud3 grr1 nth2 tgl5 yap1801 ydr348c bna3 fal1 num1 bni5 ybr238c bph1 yol057w rad6 ubp3 ecm2 bsc2 inp53 sat4 sec14 bst1 gas1 tlg1 tlg2 bub1 bub3 mad1 myo2 ism1 cdc20 mad3 ist3 kin2 tef4 cns1 yel023c cgi121 gon7 hef3 idp2 kae1 npa3 pho81 por1 rnh202 sgo1 cdc11 gsp1 bud6 smk1 bud8 bud9 rax1 oms1 bug1 bul2 upc2 bzz1 plc1 caf16 sah1 caf20 dbf2 cct2 cct3 cct6 ent2 fis1 mdv1 tcp1 ybl029w yhr001w caj1 cak1 cdc28 cdc34 efb1 hsl1 lys4 rad34 tef2 yfr016c car1 jjj1 pdc5 pfk2 gal7 mob2 sis1 vma6 cbp6 gsc2 ylf2 cce1 rad14 cch1 mid1 mpt5 sti1 ubr1 ccs1 sod1 cct7 ime2 lia1 pex7 plp2 rad28 sen2 thi3 vid27 ded81 slc1 gcn20 hgh1 wtm2 ydr128w hsp150 kcc4 dog1 dog2 met6 pyc1 rgd1 vma22 cdc123 dma2 tps1 fur1 hms1 mcr1 spe3 tsa2 vas1 rbg2 gsh1 kgd2 ymr31 yol098c ilv6 pnc1 gtt2 cdc25 mds3 rim11 ybr225w cdc6 clb4 cln1 cln3 dal7 sic1 ssc1 whi3 hom2 hsp31 thr4 ykr011c ylr356w hem15 ilv1 kic1 sem1 vps13 eap1 trp2 ydl025c ypk2 cdc4 cdc53 drs2 etr1 gdh2 mep3 met30 mms1 dhh1 npt1 rpn2 krs1 ptm1 ufd2 yos9 cdc50 pdc6 rub1 sgt1 ufo1 ydr306c yjl149w ylr352w cin1 pph21 pph22 tpd3 yck2 yrr1 zds1 ydr196c dnf1 ptc6 ubi4 ssa4 cep3 cex1 los1 vph1 ent1 ent3 sse2 swa2 vps27 yap1802 chs1 cox20 pfa4 cia1 nar1 ubp9 yhr122w rpl35a pac2 cin8 gde1 yrb2 pca1 srl3 yer138c ypl014w skm1 clb1 ymr259c cue5 dbp1 syp1 yll054c mob1 pep1 sla2 ykl069w clg1 vma10 rga1 rbg1 ydr266c ygr067c yjl144w ynl234w cmk1 cmk2 cmp2 cna1 cnb1 ede1 gad1 hch1 hsp12 hul5 ils1 mec3 pyc2 she3 she4 tvp18 ygl242c ymr315w zeo1 vph2 idh1 sfg1 pus2 ric1 emp47 erv41 glo3 pcl8 pho86 ptc3 ygl081w pnt1 dph2 ecm38 ynl157w qns1 slp1 trr1 mir1 tat1 yrb1 csr1 rad33 siw14 ted1 tpa1 vma8 ylr218c ypk1 nmd5 cse1 csf1 csi1 csn9 pci8 rri1 rri2 rpn12 tyw1 ypp1 stb3 ctr3 cue1 cue4 dsk2 cup5 ppa1 tfp3 vma21 cup9 ndc1 cwh41 cyb5 cyc1 cym1 lat1 pda1 pdx1 ras2 kar2 dak2 dal3 guf1 pma1 emi2 faa1 gly1 gph1 pro1 rib4 rpt5 gyl1 gle1 thi22 ydl086w dcd1 dcg1 rad17 ddi1 ho fcp1 ymr118c tif34 gwt1 fab1 sba1 his4 dig1 sap190 ste12 tec1 djp1 ynl311c pho84 mdj1 mei5 lem3 dnf2 pol4 dnm1 rts1 dos2 kre2 rpn4 elm1 dps1 dre2 tah18 osh2 dse1 lyp1 pdh1 pth2 yel025c dtd1 dtr1 pdx3 rad50 vma4 dut1 dys1 lys2 ymr124w mge1 nta1 scj1 ecm22 mot3 ecm25 nas6 rpn13 rpt2 ecm30 ecm31 ecm33 ecm37 glt1 prs3 yir007w yjl062w-a pro3 yjl046w egt2 sst2 eht1 emp46 emp70 pib1 end3 scd5 spf1 ydr161w exg1 mub1 pib2 scs2 ubr2 yhr138c erg1 erg26 erg9 erv46 erv29 rot2 paa1 hxt6 tom1 ssk1 mdh2 vps9 fat1 nma111 psr2 ypl206c fap1 ste18 far10 far8 far3 hfm1 vps64 iml2 trm3 tsc13 tsl1 fcy1 yct1 fes1 rer1 tsa1 spo7 flo8 mss11 fmp46 fol2 lys1 gdi1 hom3 ths1 trp5 ura1 ydr341c frs1 frs2 frt2 tel1 fus2 gpa1 msg5 gaa1 gab1 gpi17 gal2 gap1 shr3 gcd1 gcd2 mrf1 sui3 gcd10 scw4 nus1 pet9 ynl213c yhl039w gut1 mcx1 nde1 pet10 yhm2 sod2 gcr2 gcy1 gda1 pxr1 mrs6 ypt52 vps21 ypt10 ypt32 gem1 hua2 gsf2 pdc2 ykl161c ptc4 ulp1 gle2 glk1 met4 pbi2 glo2 utr2 gyp5 gna1 gnd2 mdm1 rgs2 gpb1 gpb2 gpr1 ira1 ira2 gpd1 gpi19 gpi2 gpt2 mal12 ynl134c yhr098c grs1 grx1 hap3 gsp2 mog1 ntf2 yrb30 ygk3 gto1 gto3 gua1 guk1 yig1 stt4 sec63 yjr100c gyp7 gzf3 ham1 hap5 mtq1 rhr2 ypr085c hor2 hem1 hem12 hip1 hlr1 hmf1 hmg1 pcl1 hnm1 hom6 ybr139w ura6 pin4 sgm1 tgl1 yil151c yor215c yar009c ygp1 ylr035c-a ppt1 rmd8 stp4 yel070w hsl7 rpn14 lhs1 sno4 slm3 tam41 ypl247c hxt10 ktr4 mck1 mef1 ncp1 osh6 pdr11 pho88 smy2 ssl2 ybr030w ydl203c yhr080c yhr202w hvg1 hxt5 pkh2 ykl047w yhr182w spo13 tos1 yar062w she2 trm1 pmd1 inp2 ppa2 ykr043c nfs1 isn1 mlh1 xks1 sgd1 ixr1 izh3 jid1 oac1 psd1 rtn2 ydl063c ylr426w toa1 rot1 sil1 vps52 ymr196w kel2 yer184c ybt1 sog2 yjr061w kog1 tco89 rnh203 ydr239c ypr115w lag2 lap3 pin3 sqt1 vrp1 lcb1 tsc3 lcb5 ylr326w leu2 lro1 yjl045w ylr422w yme2 ybr108w yfr039c rax2 rps23b tps3 ypt11 lys5 yor283w mal32 map1 mch1 prd1 rgp1 pup1 mdm30 sae3 mer1 rho2 vhs1 met16 met28 met31 mfb1 nup192 mkc7 ssh4 pms1 mmr1 mmt2 mnn5 neo1 pyk2 mst27 mst28 sls1 urh1 rps19a nbp2 nce102 ndl1 nem1 nma2 nte1 npr2 stp3 rpl43a nvj1 pip2 ygl039w om45 opy1 orm2 oye3 pad1 ssk22 pcl5 pkp1 pea2 pep7 spo20 vps45 pet112 pex18 pot1 pex32 pgm1 ptc5 pgs1 yap7 pgu1 pho13 pho80 rpl24b rps12 rps27a rps27b ymr050c ynl054w-b rim15 pmi40 sec66 ssh1 pmt4 png1 rho5 sfk1 pox1 ppe1 ppg1

rrd1 rrd2 ula1 rkr1 tos8 yjl132w ylr290c ycr076c ykr070w prm2 prm8 prp5 ydl124w prs1 prs2 prs5 pst1 rfs1 ycp4 rad55 ygr205w ydr186c slx5 snq2 ydr179w-a pus9 ypl183c ybr184w rav1 rav2 rho4 syg1 yjr027w rma1 rnh201 ypl191c yor021c thr1 tsc10 rpl22b vma5 spg5 spo77 yol022c ybr071w yel057c ybl054w ypr003c ykl027w uth1 sec72 ubc12 rsn1 yel1 ybp2 sae2 ylr243w ypl099c ura8 ygl140c sbe22 sga1 ylr211c sdp1 sds24 ykl091c ybr047w ynl181w ypr114w sef1 tcb2 sen34 ser1 sul2 ynl187w ylr267w ymr258c snx41 yer140w ykl037w ylr454w ypr083w zap1 zim17 ybl005w-b yel007w yjr024c ymr111c yor251c ypl158c sno2 spe4 ypl257w-b vps74 stv1 vma7 sur4 tps2 ygr066c xdj1 ypl105c tip1 tkl2 vps51 trm7 trr2 tvp15 uba3 ydr051c ypl236c yor388c ymr027w ynd1 vma9 ypt35 ylr149c ybr063c yjl206c yal049c

Cluster 34, 3 Proteins

ypl066w tus1 ycf1

Cluster 35, 3 Proteins

mbr1 pig1 yjl118w

Cluster 36, 8 Proteins

atg1 atg13 atg17 ubp11 atg29 cis1 ybr197c yjr156c

Cluster 37, 11 Proteins

smc5 gln4 ynl019c yor352w smc6 nse4 kre29 nse5 mms21 nse3 nse1

Cluster 38, 2 Proteins

sag1 ypl141c

Cluster 39, 2 Proteins

yjl070c amd1

Cluster 40, 3 Proteins

mei4 rec107 rec114

Cluster 41, 2 Proteins

ybr194w ypr152c

Cluster 42, 2 Proteins

abz2 ylr057w

Cluster 43, 7 Proteins

msn4 gef1 get3 get1 get2 grx7 yhp1

Cluster 44, 3 Proteins

kes1 pxa1 pxa2

Cluster 45, 12 Proteins

fyv10 nha1 moh1 cos3 gid7 gid8 rmd5 vid24 vid28 vid30 ydl176w yhl010c

Cluster 46, 3 Proteins

mip1 sed1 sit1

Cluster 47, 3 Proteins

cdc43 ram2 ram1

Cluster 48, 10 Proteins

mia40 trm5 pep8 vps35 cox17 cox19 vps29 muk1 vps5 vps17

Cluster 49, 549 Proteins

aap1 gud1 pep3 trs31 nhp2 acb1 ade13 fre7 nup170 q0110 sec5 rrp1 cbf5 dnl4 erb1 rps3 nyv1 pwp1 vac8 ykt6 pps1 prt1 ygl100w mis1 rpl2a rps1a pwp2 rps5 ade8 nob1 sec1 sec39 tip20 ufe1 use1 bud20 hrp1 afg2 alb1 arx1 dss4 enp1 rpp0 ded1 imd4 ski8 akr1 vti1 cic1 fun12 nog1 prp43 rei1 rlp24 tif6 sec16 rix7 sed5 apl6 apm1 rpl16b rpl19a rpl3 rpl6b rpl9a rps6a ypl249c-a ypr102c apl5 apm3 aps3 pus1 vam3 vps41 brx1 hek2 kre33 kri1 krr1 nop4 rcl1 rpl1a sda1 lsg1 mnp1 nop58 rpl12a rpl16a rpl17a rpl20a rpl28 rpl33b rpl35b rpl6a rpl8a rpl8b rpp2a rps19b rps21a rps4a sik1 sof1 utp7 ygr054w cog3 nop1 snu13 utp22 are1 mag2 rsa3 erp2 nog2 dbp10 fpr4 ipi1 ipi3 mrt4 nip7 nmd3 nop13 nop15 nop53 nop7 nop9 nsa2 nug1 nup100 rix1 rlp7 rpf2 rpl18a rpl20b rpl27a rpl30 rpl31a rpl4a rpl5 rpl7a rsa4 ydr012w enp2 rpl25 rps13 rps22a rps22b rps7a rps8a rps9b sbp1 ski3 stm1 asf2 ecm16 naf1 asi1 asi3 ynl260c rps17a snx4 utp13 atg8 atp14 sec17 tif5 yor1 atp22 rpl10 rrp9 sec15 sec8 nop2 rio2 slx9 bos1 sly1 bet3 bet5 gsg1 gyp6 kre11 trs120 trs130 trs20 trs23 trs33 ypt1 ypt31 rpl11b rps20 tsr1 bfr2 bms1 emg1 esf1 esf2 faf1 hca4 imp3 lcp5 mpp10 nan1 noc4 nop14 rps11b utp20 utp30 bim1 rpl12b rps25a rps25b rrs1 dbp3 bud21 dia2 dip2 gdt1 mrd1 nop6 rrp12 smf3 utp10 utp15 utp21 utp4 utp6 utp8 utp9

bna1 puf4 cdc48 tcb3 bpl1 jip5 rrp5 dbp9 drs1 ebp2 has1 loc1 mak11 mak21 noc2 noc3 nop12 nsa1 puf6 rpf1 rpl13a rpl15a rpl1b rpl27b rpl7b rrp15 spb4 ssf1 ytm1 btn2 snc1 snc2 yif1 gpi15 rpl13b rpl38 utp18 yer102w bud22 ygr271c-a ygr272c yke2 rpl2b rps4b imd2 dhr2 est1 gar1 kem1 nsr1 rom2 rps24b ski2 sro9 urb1 ycr016w ygr283c yhr072w-a ymr310c ypl009c cbp2 cbp3 cbp4 nup85 cbr1 rpl43b ccz1 vam6 ypt7 imp4 rpl17b sec11 cog6 dfm1 pac10 rai1 rps17b ubx3 ubx5 ubx6 ubx7 ygl108c rcy1 rps1b cha1 dbp2 ela1 mak5 mss116 nop16 rpl32 rpl33a rpl39 rps15 rps26a spb1 ssf2 fpr3 rrp14 sec31 tif35 rpl36a rps2 tub4 ynk1 cog1 cog2 cog4 cog5 cog7 cog8 gos1 spc1 dsl1 coq10 nsp1 nup49 nup57 nup82 nup133 nup145 pno1 srp40 pkc1 ypr045c utp14 oaf1 rps9a ctp1 tsr2 rli1 dbp7 dbp6 nat3 rps11a dcw1 rpl29 sso1 rpl18b rps14a rps16b rps18a tma64 lst4 dim1 ltv1 rok1 dip5 mak16 rkm2 ygl146c lys20 dse4 sec20 sec4 pol5 dyn2 sxm1 edc2 tis11 yer077c emp24 erp1 erp3 erp4 erv25 mdr1 rps14b rps24a rps28b rps7b ykl023w ydr412w rpl24a ero1 ost1 sec13 ole1 sss1 erp5 gpi10 spc2 put3 rrp3 exo70 exo84 sec3 sec6 sro7 fcf2 sas10 utp11 yil161w fap7 yhl001w fcy21 yen1 fre3 hcr1 tif11 ygr102c fun14 jhd2 vps8 rpp2b nup84 gfd2 gim3 gim4 gim5 pfd1 gis2 sft1 grc3 sfb2 ypr078c nhx1 sfa1 rtn1 ylr363w-a rps10a his3 rrb1 stt3 nup188 isa2 kex1 rps6b yil091c jjj2 kap120 nic96 rpl15b sec9 rps16a tpt1 ktr5 tfb5 leu4 leu9 yvh1 ysw1 rps23a yar1 rpl37b rpl26b met22 ncl1 rps26b mdm20 nup120 rpp1b ugp1 utp23 mrh1 rpl31b tma16 mso1 sso2 mtg1 ygl036w prs4 rpl21a rpl21b rpl26a ydr381c-a nop8 osh3 rrf1 sop4 rdr1 rmt2 nup157 vam7 ost2 ost3 ost4 ost5 ost6 swp1 wbp1 sbh1 sbh2 pam1 tcm62 pdr10 syn8 vps33 pep5 vps16 vps3 pex27 pfk27 rpl19b rpl42b rps30a ygr210c ylr003c yip3 rgt2 yae1 rps18b shm1 sec61 yer067w tma20 ybl111c yfl046w ydr545w sed4 yop1 skn1 spc3 ynr021w vts1 yor051c yor192c-c yjl147c yor059c ynl176c ybr242w

Cluster 50, 2 Proteins

dom34 hbs1

Cluster 51, 3 Proteins

fol1 ymr074c ura3

Cluster 52, 3 Proteins

inp51 irs4 tax4

Cluster 53, 10 Proteins

rpp1 pop1 pop4 pop5 pop6 pop7 pop8 rmp1 rpr2 snm1

Cluster 54, 4 Proteins

vtc1 vtc4 vtc2 vtc3

Cluster 55, 3 Proteins

mak10 mak3 mak31

Cluster 56, 91 Proteins

ynl254c rho1 leu1 cdc42 adh7 hcm1 srp68 mmf1 arg3 ygr250c lip5 avt1 doa4 bag7 srp54 srp72 bem4 sge1 bio3 pac11 ygr207c gis4 bro1 snf7 stp22 vps4 lhp1 mrp8 gic1 gic2 rsr1 msb2 rdi1 ubp13 srp21 sln1 cps1 sec65 did2 vta1 did4 vps24 din7 ymr291w dld3 nej1 prx1 yef1 dug1 dug2 dug3 erg10 hsv2 sks1 est2 est3 fum1 gal3 gal80 yfl054c snf8 vps25 his6 ylr179c hkr1 sho1 yrm1 ilv3 isu1 yfh1 msm1 srp14 trk1 yil108w ydr018c ydr374c yel043w ylr287c mvb12 srn2 pex25 pex30 sac7 sdh1 sdh2 ycr090c vps20 vps28 vps36 ybl081w vps60

Cluster 57, 3 Proteins

gor1 nft1 pet122

Cluster 58, 98 Proteins

dad2 alt1 pfk26 ame1 chl4 cse4 ctf19 ctf3 dsn1 iml3 mcm16 mcm21 mcm22 mtw1 nkp1 nkp2 okp1 apd1 spc98 pho91 arg5,6 sqs1 ask1 dad1 dad4 dam1 duo1 hsk3 spc19 spc34 atg2 mas2 bbp1 mps2 nbp1 bik1 kar9 kip2 nnf1 pkh3 stu2 ymr134w bir1 ipl1 sli15 ygr031w gts1 brn1 smc2 smc4 ycg1 ycs4 pml1 yor304c-a cnm67 sfi1 spc72 set4 ynr062c cik1 kar3 spc42 spc110 mif2 tid3 ybr233w-a nca2 spc24 mas1 dph5 nuf2 spc105 spc25 mps3 vac7 his2 hmt1 npp2 ykl215c ydl199c yer156c kar1 spc97 vik1 kin4 ymr010w ybl086c msh1 prp18 spc29 mps1 ura5 yor342c ydr532c ydl073w tgl2 ybr028c ura10

Cluster 59, 2 Proteins

bet2 bet4

Cluster 60, 2 Proteins

dia4 pus4

Cluster 61, 4 Proteins

hol1 yjr012c mmp1 plb1

Cluster 62, 2 Proteins

mdm31 mdm32

Cluster 63, 241 Proteins

abf1 glc7 srl2 rna14 rsp5 ade12 reg1 tuf1 ade16 ade17 slx1 sol1 sol2 gsy2 ubp2 prc1 aly1 ypd1 rpl14a hda1 mga2 yhl009w-b aro9 ubc13 glg2 cft1 atf2 ygr130c tyr1 ynl208w ubp7 snf4 yfr017c yor220w gal83 ngr1 bni4 bre1 lge1 tfc3 yhr149c yor365c bre2 erf2 sdc1 set1 shg1 spp1 swd1 swd2 swd3 hse1 grx3 bul1 rog1 bye1 rad4 rif1 cad1 rck1 gln1 pfs2 siz1 ubc9 gsy1 tfc7 ssm4 der1 fbp1 hda2 hmg2 hrd3 npl4 otu1 shp1 spt23 ubx2 ufd1 usa1 ynl155w hsf1 rup1 cft2 fip1 mpe1 pap1 pcf11 pta1 pti1 ref2 rna15 ssu72 ysh1 yth1 tfc4 vmr1 chl1 mpd2 clp1 tos3 rcn1 tfc6 cpr3 gdb1 cpr5 pfs1 sok2 ydr248c yhl008c yhr097c tdp1 pep4 yll029w whi2 cst9 msh4 rad57 zip1 zip2 cul3 rad7 ymr323w dap1 glg1 rtt107 ddc1 rev7 suv3 dia1 nst1 ecm21 elc1 pcl6 shr5 mrm1 met17 ynr065c yml030w fpr2 gac1 rad30 sak1 sip1 gip3 ppz2 sds22 tfc1 ypi1 gip2 gip1 ygl057c gip4 glc8 jip4 mhp1 red1 reg2 syc1 yer158c yor227w hyr1 pma2 ygl220w pcl10 hda3 oct1 nsg1 hop1 idp3 hrk1 hua1 skn7 ylr419w mxr1 ibd2 ppr1 ykl075c pim1 tfs1 ids2 yhi9 tye7 ipt1 mms2 yor289w kip1 ldb19 lsc1 lsc2 rad5 ylr408c ygr068c mek1 ppz1 ypr117w msh5 msn2 sub1 nam2 ydl173w ndt80 yol159c-a pet111 pet494 pex1 pex6 ybl010c rpp1a ppq1 sia1 psr1 yal046c rad18 rod1 rcr1 rds2 rev1 rev3 rif2 rim21 sol4 rog3 sna3 sna4 ykr018c ylr392c ypr084w ygl082w sen54 yjr098c ylr257w yll023c ykl061w ynr029c tfc8 ykr096w vab2 ydr357c ygl079w ynl086w vhs2

Cluster 64, 2 Proteins

akr2 lcb4

Cluster 65, 18 Proteins

afg3 phb1 phb2 yta12 anp1 hoc1 mnn10 mnn11 mnn9 svf1 ktr3 svp26 van1 spo14 mum2 pmu1 sma1 ypr118w

Cluster 66, 4 Proteins

oca1 oca2 ycr095c yhl029c

Cluster 67, 2 Proteins

lpe10 ygr277c

Cluster 68, 3 Proteins

fzo1 mgm1 ugo1

Cluster 69, 5 Proteins

rrn11 rrn6 rrn7 slm5 sps22

Cluster 70, 2 Proteins

ai1 taz1

Cluster 71, 2 Proteins

cbp1 pet309
